# PES1 reduces CD8^+^ T cell infiltration and immunotherapy sensitivity via interrupting ILF3-IL15 complex in esophageal squamous cell carcinoma

**DOI:** 10.1186/s12929-023-00912-8

**Published:** 2023-03-23

**Authors:** Ning Ma, Rong Hua, Yang Yang, Zhi-Chao Liu, Jie Pan, Bo-Yao Yu, Yi-Feng Sun, Dong Xie, Yan Wang, Zhi-Gang Li

**Affiliations:** 1 Department of Thoracic Surgery, Shanghai Chest Hospital, Shanghai Jiao Tong University School of Medicine, Shanghai, China; 2 CAS Key Laboratory of Nutrition, Metabolism and Food Safety, Shanghai Institute of Nutrition and Health, Chinese Academy of Sciences, University of Chinese Academy of Sciences, Shanghai, China; 3 Institute of Pathology and Southwest Cancer Center, Southwest Hospital, Army Medical University (Third Military Medical University), Chongqing, 400038 China

**Keywords:** PES1, CD8^+^ cytotoxic T lymphocytes, IL15, ILF3, PD-1

## Abstract

**Background:**

Although immune checkpoint blockade (ICB) therapy has brought survival benefits to patients with specific cancer types, most of cancer patients remain refractory to the ICB therapy, which is largely attributed to the immunosuppressive tumor microenvironment. Thereby, it is urgent to profile key molecules and signal pathways responsible for modification of tumor microenvironment.

**Methods:**

Multiple databases of esophageal squamous cell carcinoma (ESCC) were integratively analyzed to screen candidate genes responsible for infiltration of CD8^+^ T cells. Expression of pescadillo ribosomal biogenesis factor 1 (PES1) in clinical ESCC samples was examined by qRT-PCR, western blotting, and immunohistochemistry. The mechanisms of PES1 were investigated via RNA sequencing and mass spectrometry followed by immunoprecipitation and proximity ligation assay. The clinical and therapeutic significance of PES1 in ESCC was comprehensively investigated using ESCC cells and mouse model.

**Results:**

PES1 was significantly upregulated and correlated with poor prognosis in ESCC patients. PES1 knockdown decreased ESCC cell growth in vitro and in vivo and enhanced the efficacy of ICB therapy in mouse model, which was established through subcutaneous inoculation with ESCC cells. Analyses on RNA sequencing and mass spectrometry suggested that PES1 expression was negatively correlated with IL15 and ILF3 was one of the PES1-associated proteins. It has been known that ILF3 interacts with and stabilizes IL15 mRNA to increase IL15 protein level. Our data further indicated that PES1 interfered with the interaction between ILF3 and IL15 mRNA and impaired ILF3-mediated stabilization of IL15 mRNA, which eventually reduced the protein level of IL15. Interestingly, the inhibitory effect of ICB therapy boosted by PES1 knockdown dramatically antagonized by knockdown of IL15, which suppressed the tumor-infiltrated CD8^+^ T cells in ESCC. Finally, we confirmed the relationships among PES1, IL15, and CD8^+^ T cell infiltration in 10 locally advanced ESCC patients receiving ICB neoadjuvant therapy and demonstrated that ICB therapy would be more effective in those with low expression of PES1.

**Conclusions:**

Altogether, our findings herein provided novel insights on biological function and clinical significance of PES1 and suggested that high expression of PES1 could suppress ILF3-IL15 axis-mediated immunosurveillance and promote resistance to ICB through restraining tumor-infiltrated CD8^+^ T cells.

**Supplementary Information:**

The online version contains supplementary material available at 10.1186/s12929-023-00912-8.

## Introduction

Esophageal squamous cell carcinoma (ESCC) is the main pathohistological type of esophageal cancer and accounts for approximately 90% of esophageal cancer cases in China [1]. ESCC has a poor prognosis and 5-year overall survival rate is less than 40% [2, 3]. The multidisciplinary comprehensive treatment regimen, including surgery, radiotherapy, and chemotherapy, is the standard strategy to treat ESCC in clinic. Although the progression in treatment regimens bring survival benefits, the curative effects remain unsatisfactory [4–6]. Therefore, new therapeutic strategies and molecular targets are urgently needed.

Breakthrough in the field of immune checkpoint blockade (ICB) therapy has brought appreciable survival benefits to cancer patients and has also changed the treatment pattern for patients. Among the immune checkpoint inhibitors, humanized anti-PD-1/PD-L1 and CTLA4 monoclonal antibodies have shown promising therapeutic outcomes [7]. Ipilimumab, targeting CTLA4, is the first immune checkpoint inhibitor approved for the treatment of patients with advanced melanoma [8]. Pembrolizumab and Nivolumab, which target PD-1, have been applied to patients with melanoma and non-small cell lung cancer (NSCLC) with an objective response rate (ORR) of 40–45% [9–11]. Immunotherapy was successfully approved as the first-line treatment of advanced-metastatic esophageal cancer [12–14], a perioperative treatment strategy for esophageal cancer based on immunotherapy is being widely investigated. The CheckMate 577 trial has shown that adjuvant therapy of nivolumab significantly improved the disease-free survival of locally advanced esophageal cancer patients who had residual pathological disease following neoadjuvant chemoradiotherapy [15]. Recently, multiple exploratory studies have demonstrated that ICB as neoadjuvant therapy, such as neoadjuvant ICB plus chemotherapy or neoadjuvant ICB plus chemoradiotherapy, may have promising results in patients with locally advanced ESCC [16–19]. However, it should be noted that immunotherapeutic responses vary among individuals, only a small population of the patients shows responds to ICB therapy. Accumulating evidence suggests that infiltration of immune cells predicts patients’ response to ICB therapy [20, 21]. Especially, CD8^+^ cytotoxic T lymphocytes (CTL) plays a central role in immunotherapy-induced tumor immunity and the infiltration of CD8^+^ CTL is positively correlated with the efficacy of ICB therapy [22, 23]. Recent studies have further suggested that patients with more infiltrating T cells have a longer overall survival than patients with low infiltration [24, 25]. Hence, reshaping the tumor microenvironment to boost CD8^+^ CTL infiltration is a promising way to enhance therapeutic efficacy of ICB.

The infiltration of CD8^+^ CTL is regulated by various factors in tumor microenvironment from immune cells, stromal cells, and tumor cells. Recently, an increasing number of studies have reported that tumor-intrinsic oncogenic signaling pathways contribute to the modification of the tumor microenvironment, leading to tumor escape from immunosurveillance and resistance to ICB [26–28]. Tumor cells can utilize the oncogenes to construct an immune suppressive microenvironment to repress T cell-mediated anti-tumor effects [29]. Thus, successful identification of these genes will lead to new therapeutic strategies; however, such studies have not yet been performed so far in ESCC cells.

Pescadillo ribosomal biogenesis factor 1 (PES1) is a nuclear protein composing of 588 amino acids. It is highly conserved in evolution and contains the C-terminal (BRCT) domain of breast cancer-related gene 1 (BRCA1) [30]. PES1 is overexpressed in a variety of solid tumors, such as liver cancer, breast cancer, pancreatic cancer, colon cancer and so on [31–33]. Elevation of PES1 results in tumor cell proliferation and malignant transformation and the poor prognosis in multiple types of cancers. However, whether PES1 can remodel the tumor microenvironment to affect ICB efficacy remains to be determined. Here, we established a subcutaneous tumor model using murine ESCC cells with PES1 knockdown and observed that PES1 knockdown increased the infiltration of CD8^+^ T cells into the ESCC subcutaneous tumors. We further demonstrated that PES1 interfered with the interaction between ILF3 and *IL15* mRNA to reduce *IL15* mRNA stability and gene expression. Moreover, PES1 knockdown enhanced the efficacy of ICB therapy. Hence, our findings herein explained the function of *PES1* in promoting ESCC progression from the perspective of immune microenvironment remodeling.

## Materials and methods

### Human ESCC samples

The paired ESCC specimens and tissue microarrays (cohort-60 and cohort-230) were collected from the Shanghai Chest Hospital and the details on cohort-60 and cohort-230 in Additional file 2: Dataset S1. We also surgically collected ESCC samples from 10 ESCC patients treated with neoadjuvant immunotherapy from a registered phase II clinical trial (ChiCTR1900026240, J Immunother Cancer 2022;10(3):e004291). Then, these patients were subjected to anti-PD-1 treatment and classified into pathological complete response (pCR) group and non-Pathological complete response (non-pCR) group at endpoint of treatment. The study was approved by the Institutional Review Board of Shanghai Chest Hospital, Shanghai Jiao Tong University.

### Animal studies

C57BL/6 J mice (male, 4 or 6 weeks old) were purchased from Gempharmatech Co, Ltd. The mice were randomly separated into designated into groups by an independent person. We used 6 or 4 mice per experimental group for all the animal experiments. Control and AKR cells (3 × 10^6^) with PES1 knockdown were injected subcutaneously into each site. For anti-PD1 treatment, when the above mice were subcutaneously injected with AKR cells for 7 days, injection of anti-PD1 (*InVivo*MAb anti-mouse PD-1 (CD279), BE0273, BioXcell, 2 mg/kg body weight) was performed every 3 days until sacrifice. The tumor size was measured every other day with a Vernier caliper. The tumor volume was calculated according to the following formula: Volume = (length × width^2^)/2. The animal study was carried out in accordance with approved protocol.

### Cell lines

The mouse esophageal squamous cell carcinoma cell line AKR [34, 35] was purchased from Shanghai BinSui Biological Technology Co., Ltd, and the 293T, EC9706 [36–39] and KYSE150 cell lines were kindly provided by Dong Xie (Chinese Academy of Science). The AKR and 293 T cell lines were cultured in DMEM, 10%FBS and 1% penicillin–streptomycin. EC9706 and KYSE150 cell lines were cultured in RPMI 1640 supplemented with 10%FBS and 1% penicillin–streptomycin. All were maintained in a humidified chamber with 5% CO_2_ at 37 ℃.

### qRT-PCR

Total mRNA was isolated from cultured cells or tumor samples using TRIzol reagent (Invitrogen), according to the manufacturer’s instructions. 1ug of RNA was then reverse transcribed into cDNA using a HiScript III 1st Strand cDNA Synthesis Kit (Vazyme, R312) according to the manufacturer’s instructions. Taq Pro Universal SYBR qPCR Master Mix (Vazyme, Q712) was used to quantify the PCR amplification. Expression levels were calculated using the ΔΔCt-method. The primers as follows:

Human-*PES1*-F: GGCCACCAACTACATCACCC

Human-*PES1*-R: AGAATGCACAGCCGCCTAAA

Human-*IL15*-F: ACCATAGATTTGTGCAGCTGTTT

Human-*IL15*-R: GCTGTTACTTTGCAACTGGGG

Mouse-*IL15-F*:CATCCATCTCGTGCTACTTGTG

Mouse-*IL15-R*: GCCTCTGTTTTAGGGAGACCT

### RNA-sequencing

RNA-sequencing was used to compare shPES1 and SCR KYSE150 cells. Total RNA was extracted using the TRIzol reagent (Invitrogen) according to the manufacturer’s protocol. Libraries were constructed using the TruSeq Stranded mRNA LT Sample Prep Kit (Illumina, San Diego, CA, USA) according to the manufacturer’s instructions. The libraries were sequenced on an illumina HiSeq X Ten platform and 150 bp paired-end reads were generated. Transcriptome sequencing and analysis were conducted by OE Biotech Co., Ltd. (Shanghai, China).

### Western blotting

HEK293T cells, KYSE150 cells and EC9706 cells were washed twice with ice-cold PBS, and lysed in RIPA buffer with protease inhibitors for 15 min on ice, after centrifugation at 13000 rpm for 10 min at 4 °C. Protein concentration was determined by Broadford. Next, the protein lysates were separated using 8% and 12% SDS-PAGE gels and then transferred to PVDF membranes (0.45 μm). After 60 min, the membranes were blocked in 5% BSA, they were incubated overnight at 4 °C with primary antibodies and then for 70 min at room temperature with the mouse and rabbit secondary antibodies. Protein levels were quantified using a Tanon-5200 multifunctional chemiluminescence instrument and normalized GAPDH levels. The antibodies used in this study are listed in Additional file 1: Table S5.

### In situ proximity ligation assay (PLA)

In situ PLAs were performed using Duolink in situ reagents (Sigma-Aldrich: DUO92102) according to the manufacturer’s recommendation. Briefly, paraffin sections were deparaffinized, antigen retrieved, permeabilized with Triton X-100 and treated with hydrogen peroxide and blocked at 37 ℃ for 1 h. Next, the primary antibodies were incubated with PES1 (mouse) and ILF3 (rabbit) overnight. Then, wash the tablet and incubated probe at 37℃ for 1 h. Ligation reaction with ligase which provided in the kit at 37 ℃ for 30 min. Amplification reaction according to the instructions at 37 ℃ for 100 min. Finally, the slides were mounted with Duolink ® in situ mounting medium containing DAPI. Imaging was performed after 15 min by using an all-in-one Fluorescence Microscope (BZ-X800, KEYENCE). The in situ PLA experiment was broadly used to detect protein–protein interaction. In the current study, total 12 clinical samples were subjected to PLA assay and positive signals were consistently detected in all samples despite the different interaction levels in these samples.

## Plasmid construction

The full-length region of human PES1 was cloned into the p23-3xFlag-GFP vector, while the coding sequence of ILF3 was inserted into the pCDH-HA-RFP vector. The fragments of PES1 and ILF3 were obtained by PCR amplification. The primer as follows:

PES1-Forward: CGGGGTACCATGGGAGGCCTTGAGAAGAAG

PES1-Reverse: TGCTCTAGAGGCTCCGGCCTTGCCTTCTTGGCCTTC

ILF3-Forward: ATTACGCTGCTAGCGAATTCATGCGTCCAATGCGAATTTT

ILF3-Reverse: ATCCTTGCGGCCGCGGATCCCTAGGAAGACCCAAAATCATGATAGC

The shRNAs targeting PES1 were designed with the help of the GPP Web Portal website and cloned into the pLKO.1-puro vector. The primers as follows:

Human-PES1-shRNA-forward sequence:

5’CCGGCACATCATCAAGGAACGGTATCTCGAGATACCGTTCCTTGATGATGTGTTTTTG3’

Human-PES1-shRNA-reverse sequence:

5'AATTCAAAAACACATCATCAAGGAACGGTATCTCGAGATACCGTTCCTTGATGATGTG3'

Mouse-PES1-shRNA-forward sequence:

5'CCGGCGAGAGTACAAGGTGTTTGTTCTCGAGAACAAACACCTTGTACTCTCGTTTTTG3'

Mouse-PES1-shRNA-reverse sequence:

5'AATTCAAAAACGAGAGTACAAGGTGTTTGTTCTCGAGAACAAACACCTTGTACTCTCG3'

IL15-shRNA-forward sequence:

5'CCGGCAGTGCTACTTGTGTTTACTTCTCGAGAAGTAAACACAAGTAGCACTGTTTTTG3'

IL15-shRNA-reverse sequence:

5'AATTCAAAAACAGTGCTACTTGTGTTTACTTCTCGAGAAGTAAACACAAGTAGCACTG3'

### Immune profiling by flow cytometry

To quantify immune cells, single-cell suspensions were prepared from fresh tumor tissues by using the Tumor Dissociation Kit (MACS Miltenyi Biotec, 130-096-730), according to the manufacturer’s instructions. Then, anti-CD45, anti-CD3, anti-CD8, anti-NCR1, anti-CD4, anti-GZMB and anti-F4/80 were added in the single-cell suspensions for 20 min. The cells were then washed and resuspended in PBS (including 2% FBS). All samples were run on a BD FACS Verse flow cytometer and analyzed by FlowJo_V10 software. The information of antibody was described in Additional file 1: Table S5.

### RNA binding protein immunoprecipitation assay (RIP assay)

The Magna RIP™ Kit (17-700; EMD Millipore, Billerica, MA, USA) was used for the RIP assay according to the manufacturer’s protocol. The steps are as follows: Firstly, prepare complete RIP lysis buffer and keep prepared buffer on ice. Harvest cells by first washing the cells twice with ice-cold PBS, then collect cells by centrifugation at 1500 rpm for 5 min at 4℃ and discard the supernatant. Re-suspend the cell pellet in an equal pellet volume of complete RIP lysis buffer. Mix by pipetting up and down until the cells have been dispersed and the mixture appears homogeneous. Incubate the lysate on ice for 5 min. Dispense the lysate into ~ 200ul aliquots in nuclease-free microcentrifuge tubes. Secondly, transfer 50ul of magnetic bead suspension to each tube. Wash the magnetic beads twice with 0.5 mL of RIP wash buffer using a magnetic separator. Add the antibody (anti-ILF3) to the appropriate tube of magnetic beads. Incubated with rotation for 30 mintutes at room temperature. Wash the beads twice with 0.5 mL of RIP wash buffer using a magnetic separator. Thirdly, prepare the RIP immunoprecipitation buffer and place the tube from the second on a magnetic separator and discard the suspernatant. Re-suspend the pellet in each tube in 900ul of RIP immunoprecipitation buffer. Thaw the RIP lysate from section one quickly and centrifuge at 14,000 rpm for 10 min at 4 ℃ and add to each tube containing the beads-antibody complex in RIP immunoprecipitation buffer. Incubate all the tubes by rotating at 4 ℃ for overnight. Centrifuge the immunoprecipitation tubes briefly and place on a magnetic separator and discard the supernatant. Wash the beads a total of six times with 500 µl of ice-cold RIP wash buffer using a magnetic separator. Finally, RNA was isolated and extracted by using TRIzol reagent (Invitrogen), according to the manufacturer’s instructions, and the level of IL15 mRNA in the RIP complex was assayed by using qRT-PCR, results were analyzed using ΔΔCT method.

### Immunohistochemistry (IHC)

Firstly, put the removed tumor tissue or ESCC samples into 4% formalin for fixation. After dehydration and waxed, the tumor sections were embedded in paraffin. Cut the tissues into 5um slides. The slides were incubated with primary antibodies: PES1, IL15, CD8, CD3, GAMB, NCR1, F4/80 and CD4, the specific steps were performed using the GeneTech (Shanghai) Company Limited (GK500710). The information of antibody was described in Additional file 1: Table S5. The staining method of tissue microassay (TMA) is also consistent. The extent of protein expression (PES1/IL15/CD8) were automatically scored by the Vectra 2 system (PerkinElmer). The staining of PES1 and IL15 were interpreted using the H-score, the CD8 was using 2-positivity. For the PES1 expression, the tissues with a final PES1 H-score < 130 were defined as low expression and those with a final score ≥ 130 were defined as high expression. Survival curves were constructed using the Kaplan–Meier method and analyzed by the log-rank test.

### IP and mass spectrometry

3 × Flag-tagged protein IP was performed according to the anti-Flag M2 manual. Specifically, EC9706 cells transfected with 3 × Flag-PES1 and control vectors were lysed in IP lysis buffer (50 mM Tris–HCl, PH = 7.4 with 150 mM NaCl, 1 mM EDTA, and 1% Triton X-100) with protease inhibitors for 60 min on ice. After centrifugation at 13,000 rpm for 20 min at 4 °C, supernatants were taken out to other tubes and incubated with anti-Flag M2 beads on a rotator overnight in refrigeration house. After incubation, the beads were pelleted and washed by TBS (50 mM Tris–HCl, 150 mM NaCl, PH = 7.4) for five times. Elute with Flag peptides for 1 h. The eluate was resolved by SDS-PAGE western blot. Coomassie brilliant blue staining was performed as its protocol. Differential bands were disposed with mass spectrometry analysis.

### Statistical analyses

All data were analyzed using GraphPad Prism version 7. Quantitative values are presented as the mean ± S.E.M. Statistical differences between two experimental groups were analyzed using the t test. Statistical significance was set at *p* < 0.05.

## Results

### PES1 was highly expressed and negatively correlated with tumor-infiltrating CD8^+^ CTL in ESCC

**Fig. 1:**
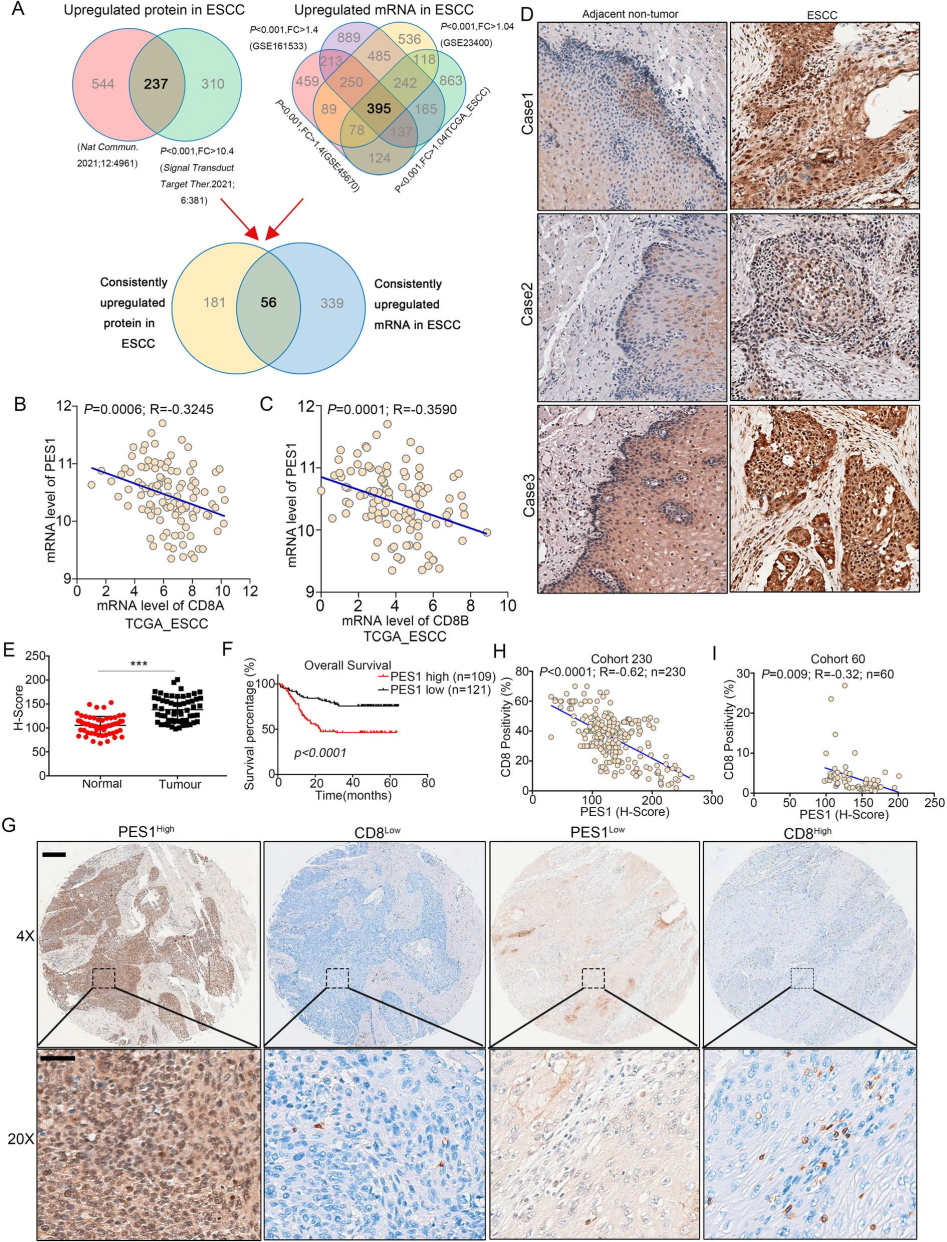
The expression of PES1 is upregulated and negatively correlated with CD8^+^ CTL infiltration in ESCC. **A** Data sources used in study and the process of gene screening. **B**, **C** Correlations between the expression of PES1 and the expression of CD8A and CD8B. Correlation was estimated using TCGA_ESCC data. **D** IHC staining of PES1 in ESCC tumor and paired nontumor tissues. Scale Bar = 100 μm. **E** Scatter diagram of PES1 staining score in the tissue of the 60 paired ESCC samples. ***p < 0.001. **F** Kaplan–Meier analysis of overall survival of tissue microarray (TMA) data containing 230 patients. **G**–**I** The expression of PES1 and CD8 in the ESCC tissue array examined and scored by IHC. The representative images from Cohort 230 were shown in panel **G** and the Pearson correlations between their expressions were shown in panel H for Cohort 230 and panel I for Cohort 60. Scale Bar = 200 μm (upper) and 50 μm (lower)

We hypothesized that the genes responsible for CD8^+^ CTL infiltration restriction are upregulated during the development and progression of ESCC. To screen candidate genes, we comprehensively analyzed six ESCC datasets, including four mRNA expression datasets (GSE161513, GSE23400, GSE45670, and TCGA_ESCC) and two proteomics datasets [40, 41]. The analysis identified 56 genes significantly upregulated at both mRNA and protein levels in ESCC (Fig. 1A and Additional file 1: Table S1), and interestingly, Pearson correlation analysis using TCGA_ESCC dataset revealed that PES1 expression assumed the strongest negative correlation with the levels of CD8A and CD8B, two markers of CD8^+^ CTL (Fig. 1B, C) (Additional file 1: Tables S2 and S3), which promoted us to focus on PES1 for further investigation.

First, we validated the expression of *PES1* and the correlation between PES1 and CD8^+^ CTL using our own ESCC tissue samples. We collected 16 pairs of fresh ESCC tissue samples and matched non-tumor tissues for quantitative RT-PCR (qRT-PCR). The results indicated that the mRNA level of *PES1* was significantly higher in ESCC tissues than that in non-tumor tissues (Additional file 1: Fig. S1A), which was consistent with the analysis on public databases (Additional file 1: Fig. S1B–D). We also examined the protein expression of *PES1* in another 12 pairs of fresh ESCC tissues and matched non-tumor tissues and proved that the protein level of *PES1* was dramatically upregulated in ESCC samples compared with their non-tumor counterparts (Additional file 1: Fig. S1E). Additionally, increased protein levels of *PES1* were observed in ESCC samples by immunohistochemistry (IHC) in 60 pairs of paraffin ESCC samples and matched non-tumor tissues (Cohort-60) (Fig. 1D, E). To explore the clinical significance of *PES1* in ESCC, an ESCC tissue microarray containing 230 tumor specimens (Cohort-230) was subjected to IHC analysis. The results showed that patients with high PES1 expression had worse prognosis than those with low PES1 expression (Fig. 1F). After adjusting for clinical factors including tumor stage, we found that higher PES1 expression was a prognostic risk factor (HR 3.17 and *P* < 0.001) (Additional file 1: Fig. S1F) but there were no correlations between PES1 expression and age, gender, tumor grade or TNM stage (Additional file 1: Table S4). We further evaluated the relationship between PES1 and CD8^+^ CTL in ESCC using the two ESCC tissue cohorts. In Cohort-230, the data confirmed a significantly negative correlation between the protein levels of PES1 and CD8 (Fig. 1G, H), which was further confirmed by IHC staining in Cohort-60 (Fig. 1I). Taken together, the expression of PES1 was upregulated in ESCC and was negatively correlated with patient prognosis and CD8^+^ CTL infiltration.

### Expression deficiency of PES1 promoted CD8^+^ CTL infiltration in ESCC

**Fig. 2:**
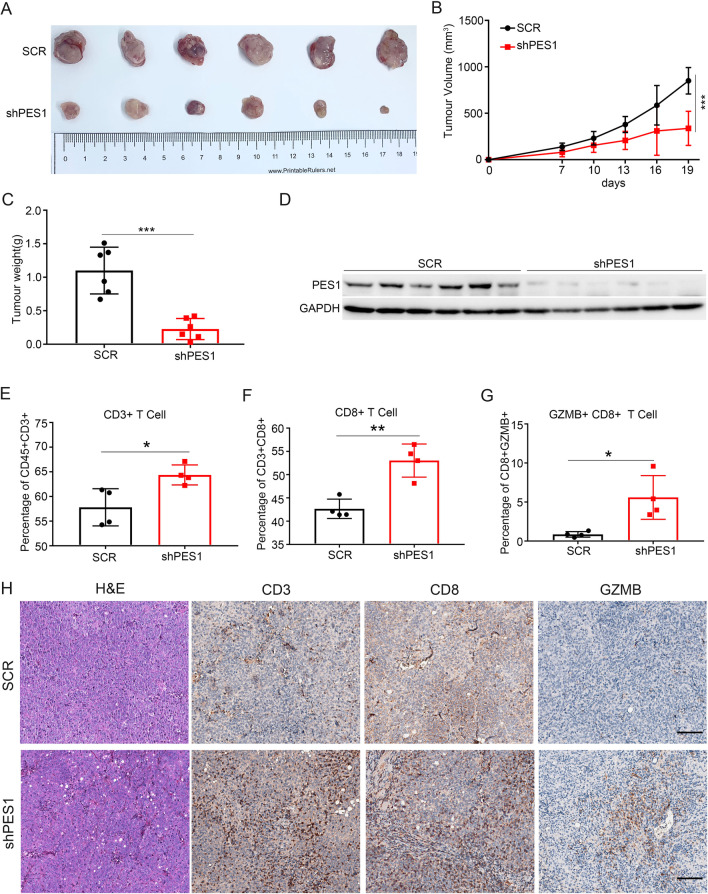
PES1 promotes ESCC tumorigenesis and inhibits CD8^+^ T cell infiltration. **A**–**C** A subcutaneous tumor formation assay in C57BL/6 J mice was performed to evaluate the effects of PES1 knockdown on AKR cells. (n = 6 mice per group) The representative image of subcutaneous tumors (**A**), tumor growth curve was shown **B** and tumor weight were shown (**C**). ***p < 0.001. **D** Western blot analysis PES1 expression in subcutaneous tumors. **E**–**G** Effect of PES1 knockdown in AKR tumors on T cell function. Representative quantification of CD45^+^/CD3^+^ T cells (**E**), CD3^+^/CD8^+^ T cells **F** and CD8^+^/GZMB^+^ T cells **G** for the indicated groups (n = 6 mice per group). *p < 0.05; **p < 0.01. **H** HE and IHC were performed to examine the tumor area and detect the expression of CD3, CD8, and GZMB. Scale bar = 100 μm

Since PES1 was negatively correlated with CD8^+^ CTL infiltration, we explored whether PES1 was involved in the regulation on the tumor immune response. For this purpose, AKR cells, a mouse ESCC cell line, were stably infected with lentivirus of scramble shRNA (SCR) or PES1-knockdown shRNA (shPES1) and subcutaneously inoculated into immunocompetent C57BL/6 J mice. Knockdown of *PES1* remarkably reduced the growth of AKR subcutaneous tumors in vivo (Fig. 2A) as indicated by the measurement on tumor volume and tumor weight (Fig. 2B, C). Western blotting confirmed the knockdown effect of *PES1* in subcutaneous tumor masses (Fig. 2D). To determine the involvement of the immune system, we tested the growth by MTT and colony formation in PES1-overexpression and control EC9706 cells and PES1-knockdown and control KYSE150 cells. Then, we inoculated PES1-knockdown and control KYSE150 cells into nude mice. Our results showed that PES1 had no effect on tumor growth in cell lines and nude mice (Additional file 1: Fig. S2A–F). Therefore, we speculated that the function of PES1 required complete microenvironment, which promoted us to examine the potential involvement of PES1 in distribution of immune cells in ESCC tissues. To this aim, we analyzed a panel of tumor-infiltrating immune cells, including CD4^+^ T lymphocytes, CD8^+^ CTL, NK cells, and macrophages, by flow cytometry (Additional file 1: Fig. S3A). As expected, PES1 knockdown dramatically enhanced the infiltration of CD8^+^ CTL in AKR subcutaneous tumors (Fig. 2E, F) and also increased the percentages of GZMB^+^/CD8^+^ CTL, a marker of activated CD8^+^ CTL, in tumor microenvironment compared to SCR group (Fig. 2G). However, the infiltration of CD4^+^ T lymphocytes, NK cells, and macrophages had no significant difference between AKR/shPES1 and AKR/SCR (Additional file 1: Fig. S3B–D). IHC staining on tumor tissues from subcutaneous tumors indicated the elevations of CD3, CD8, and GZMB signals in subcutaneous tumors of AKR/shPES1 compared with that of AKR/SCR (Fig. 2H). However, markers of other immune cells were not altered between the two kinds of subcutaneous tumors (Additional file 1: Fig. S3E). Therefore, the result suggested that PES1 knockdown facilitated CD8^+^ CTL infiltration in ESCC tissues.

### PES1 inhibited CD8^+^ CTL infiltration through reducing IL15 expression

**Fig. 3:**
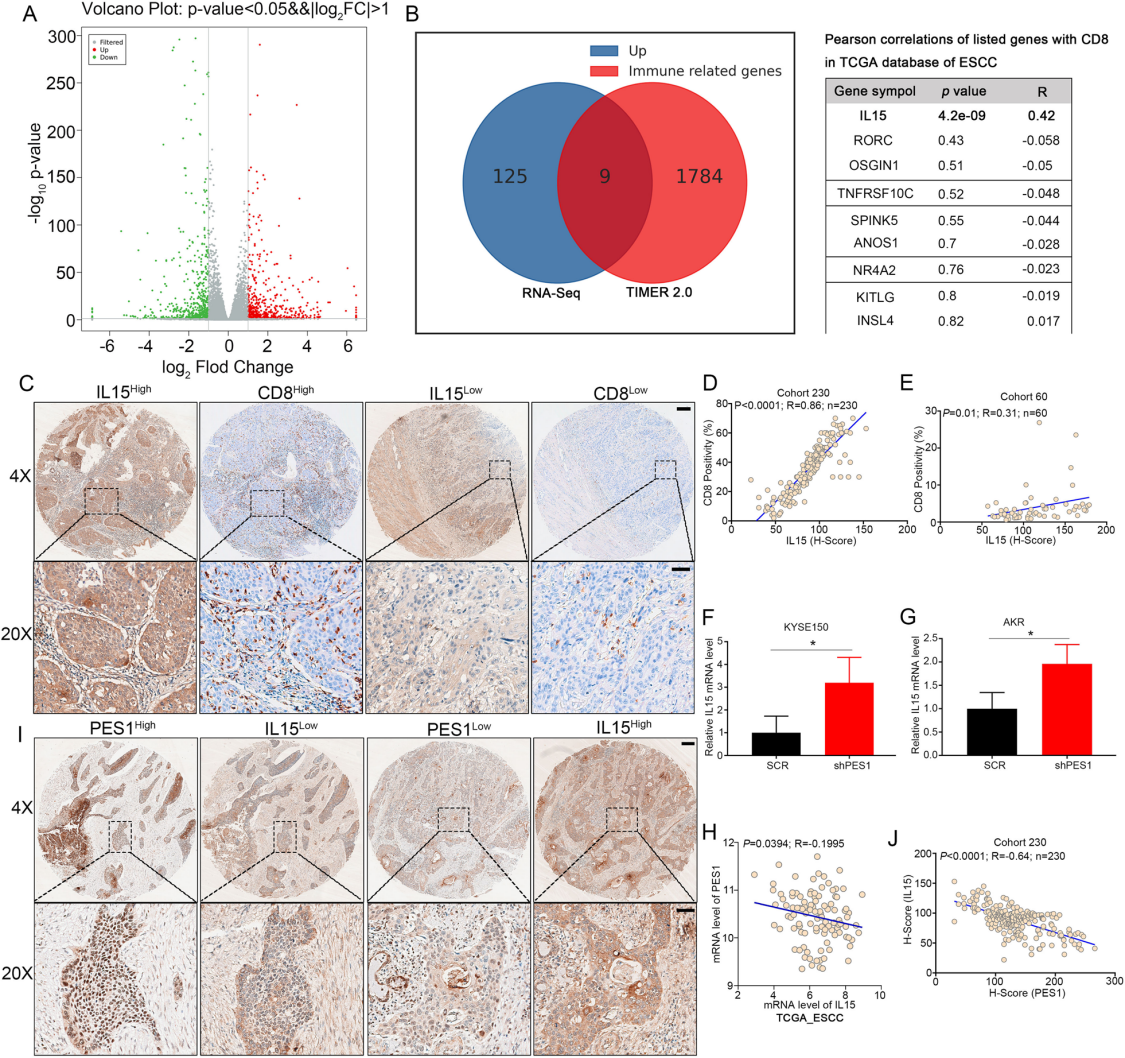
Knockdown of PES1 inhibits the expression of IL15. **A** Volcano map showing the up and down genes in shPES1 and SCR KYSE150 cells. **B** The process of screening the immune related genes. The immune related genes were from TIMER2.0 database. The correlation between IL15 and CD8 using TCGA database of esophageal cancer. **C–E** The expression of IL15 and CD8 in the ESCC tissue array was examined by IHC and scored. The representative images were shown in (**C**), and the correlations between their expression were analyzed (**D**, **E**). Scale Bar = 200 μm (upper) and 50 μm (lower). **F**–**G** Effect of PES1 knockdown on IL15 transcription in KYSE150 (**F**) and AKR (**G**) cells (n = 3 independent experiments). *p < 0.05. **H** Correlation between PES1 expression and IL15 expression in TCGA_ESCC database. **I**, **J** Correlation between PES1 expression and IL15 expression in the ESCC tissue array. The representative IHC images were shown in (**I**), and the correlations between their expression were analyzed (J). Scale Bar = 200 μm (upper) and 50 μm (lower)

Furthermore, we explored the mechanism by which PES1 affected CD8^+^ CTL infiltration. RNA-Seq was performed using KYSE150 ESCC cell line transfected with SCR or shPES1 to profile significantly altered genes by PES1 knockdown (Fig. 3A and Additional file 3: Dataset S2). To identify candidate genes responsible for immune regulation, we used Venn diagram analysis to screen the overlayed genes between the upregulated genes by PES1 knockdown in KYSE150 cells and the immune-related genes from TIMER2.0 database, which revealed nine candidate genes (Fig. 3B). Interestingly, only mRNA level of *IL15* was positively correlated with that of CD8A and CD8B using TCGA_ESCC (Fig. 3B). IHC results from both Cohort-230 and Cohort-60 consistently indicated that IL15 protein level was significantly associated with CD8 protein level (Fig. 3C–E). Actually, *IL15* has been reported to be correlated with the infiltration of CD8^+^ CTL [42], implying *IL15* as a potential downstream target for PES1.

Indeed, we confirmed through qRT-PCR that the mRNA level of *IL15* was higher in ESCC cells transfected with shPES1 than in those transfected with SCR (Fig. 3F, G). Consistently, TCGA_ESCC analysis suggested that PES1 expression was negatively correlated with the IL15 expression (Fig. 3H). In addition, the protein level of IL15 and the IL15 secretion were also increased in KYSE150 and AKR cells with PES1 knockdown (Additional file 1: Fig. S4A–D), which was further confirmed in subcutaneous tumor samples via western blotting (Additional file 1: Fig. S4E). In contrast, the mRNA and protein levels of *IL15* were decreased in EC9706 cells with PES1 overexpression compared to empty vector control (Additional file 1: Fig. S4F and S4G). Furthermore, we examined the protein levels of PES1 and IL15 in the two ESCC cohorts by IHC and inverse correlation between protein levels of PES1 and IL15 was consistently observed (Fig. 3I, J, and Additional file 1: Fig. S4H). Moreover, patients with PES1^low^/IL15^high^ showed significantly prolonged overall survival time compared to those with PES1^high^/IL15^low^ (Additional file 1: Fig. S4I). Collectively, PES1 might inhibit the expression of *IL15* in ESCC and have inverse prognostic significance with *IL15*.

### IL15 was required for enhanced CD8^+^ CTL infiltration in ESCC with PES1 deficiency

**Fig. 4:**
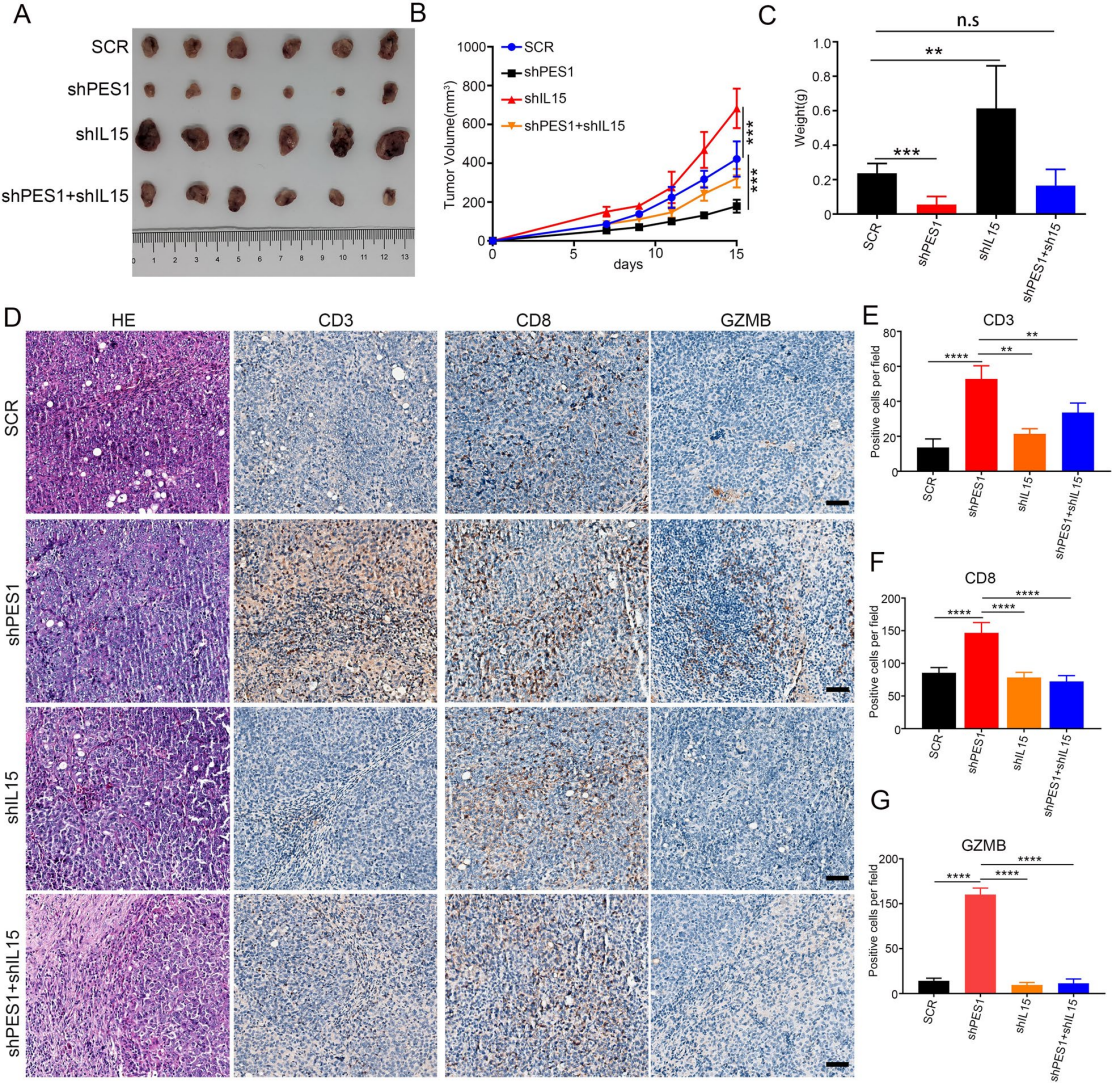
PES1 inhibits T cell infiltration by weakened the expression of IL15. **A** The representative image of subcutaneous tumors in indicated group. **B**, **C** Tumor growth curves **B** and tumor weight **C** of the indicated groups (n = 6 mice per group). Statistical analysis by t test: *p < 0.05; **p < 0.01. **D** HE and IHC were performed to examine the tumor area and detect the expression of CD3, CD8, and GZMB. Scale bar = 100 μm. **E**–**G** The CD3 + T cells (**E**), CD8 + T cells (**F**), GZMB + CD8 + T cells **G** were quantified. n = 6. Data shown as mean ± SD, statistical analysis by t test: *p < 0.05; **p < 0.01; ***p < 0.001; ****p < 0.0001

We next asked whether IL15 expression contributed to increased CD8^+^ CTL infiltration in ESCC with *PES1* deficiency. For this purpose, we functionally knockdown *IL15* in AKR cells by shRNA. Intriguingly, *IL15* knockdown significantly promoted the growth of subcutaneously inoculated ESCC cells with SCR (Fig. 4A–C). Only *PES1* knockdown decreased tumor volume and weight by 72% and 85% (*vs.* SCR), respectively. However, with *IL15* knockdown and *PES1* knockdown have no reduction in tumor volume and weight (Fig. 4A–C). Additionally, IHC staining using the aforementioned samples demonstrated that PES1 knockdown promoted the infiltration and activation of CD8^+^ CTL as indicated by the increased percentage of cells with CD8^+^ and GZMB^+^, respectively (Fig. 4D–G). However, these effects were abrogated by *IL15* knockdown (Fig. 4D–G). Therefore, our results suggested that PES1 might inhibit CD8^+^ CTL infiltration in ESCC by impeding the expression of *IL15*.

### PES1 interacted with ILF3 to regulate IL15 expression

**Fig. 5:**
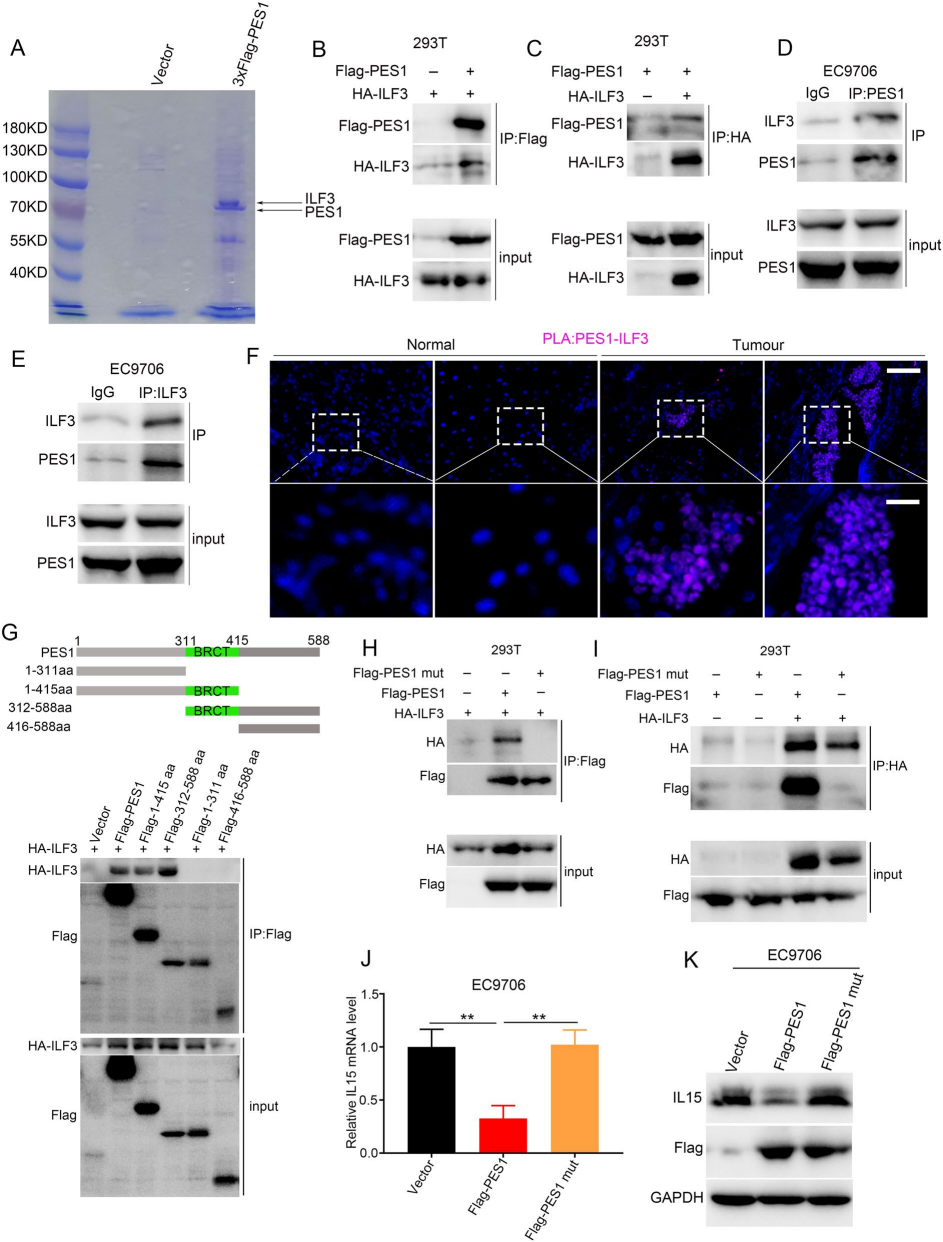
PES1 interacts with ILF3. **A** Immunoprecipitation with anti-Flag M2 beads in EC9706 cells followed by coomassie brilliant blue staining assay, arrow indicates PES1 and ILF3. **B**, **C** Co-immunoprecipitation assay in 293 T cells co-transfected with Flag-PES1 and HA-ILF3. **D**, **E** Examination of the interaction between endogenous PES1 and ILF3 by co-immunoprecipitation in EC9706 cells. **F** Representative PLA images detecting the interaction between endogenous PES1 and ILF3 in normal and tumour tissues. Scale Bar = 100 μm (upper) and 50 μm (lower). **G** Co-IP was used to identify the ILF3 binding site in PES1. The full-length PES1 and deletion mutant constructs were shown. PES1, ILF3 and their corresponding deletion mutants were co-transfected into 293 T cells. 48 h later, the cells were collected for Co-IP. **H**, **I** 293 T cells were transfected with control vector and PES1, PES1 mut, HA-ILF3 plasmid, respectively. After 48 h, co-immunoprecipitation assay was performed. **J**, **K** The expression of IL15 in EC9706 cells was examined using qPCR and western blots. **p < 0.01

To further delineate the mechanism by which PES1 regulated *IL15* expression, we profiled PES1 interacting proteins by co-immunoprecipitation followed by mass spectrometry identification. In brief, EC9706 cells were transfected with 3 × Flag-PES1 or empty vector as control. 3 × Flag-PES1 was immunoprecipitated from cell lysates with anti-Flag-M2 beads, and the complex were separated by SDS-PAGE followed by Coomassie blue staining. Several unique bands were detected in the group of 3xFlag-PES1 but not in the control (Fig. 5A and Additional file 4: Dataset S3). These bands were then subjected to tandem mass spectrometry for protein identification. Several known PES1-associated proteins were detected, such as BOP1, WDR12 and DDX27[43], suggesting the reliability of our mass spectrum data. Among the PES1-assocaited proteins, we noticed two interleukin expression regulators, ILF2 and ILF3, which have been known to stabilize mRNA and thus increase gene expression [44, 45].

To validate the interaction between PES1 and ILF2 or ILF3, we transfected EC9706 cells with 3 × Flag-PES1 and performed IP with Flag antibody, and observed that PES1 could interact with ILF3 but not ILF2 (Fig. S5). The interaction between PES1 and ILF3 was also observed in 293 T cells (Fig. 5B, C). Furthermore, the interaction between endogenous PES1 and ILF3 was detected in EC9706 cells (Fig. 5D, E). Using in situ proximity-ligation assay (PLA), a method that enables the exhibition of close protein–protein interaction (< 40 nm) while preserving the structural integrity of the cells [46], we found a tight association between PES1 and ILF3 in nuclei of paraffin sections, and the interaction was much stronger in ESCC tissues than non-tumor counterparts (Fig. 5F). Then, we constructed subclones of PES1 as indicated in Fig. 5G to identify fragments of PES1 responsible for the interaction of PES1 with ILF3. Through co-expression of different PES1 fragment with ILF3 in 293 T cells, we noticed that the BRCT domain of PES1 was required for the interaction between PES1 and ILF3 (Fig. 5H). To confirm this finding, we transfected 293 T cells with ILF3 and wild-type PES1 or point mutant PES1 W397R (PES1 mut), a critical residue within the BRCT domain [30] and found that PES1, but not PES1 mut, could interact with ILF3 (Fig. 5H, I). Thus, PES1 interacted with ILF3 via the BRCT domain. Moreover, qPCR and western blotting results showed that wild-type PES1 decreased the level of IL15, whereas PES1 mut barely affected the IL15 expression (Fig. 5J, K), implying that the interaction between PES1 and ILF3 was critical for IL15 expression. To verify whether ILF3 participated in the IL15 regulation by PES1, we exogenously expressed ILF3 in ESCC cells and noticed that ILF3 overexpression enhanced the mRNA and protein levels of IL15, which could be alleviated by PES1 expression. However, PES1 mut could not interfere with the ILF3-induced IL15 upregulation (Additional file 1: Fig. S6A, B). Taken together, the interaction between PES1 and ILF3 might be required for the PES1-induced downregulation of IL15 in ESCC cells.

### PES1 disrupted ILF3-IL15 mRNA interaction and reduced IL15 mRNA stability

**Fig. 6:**
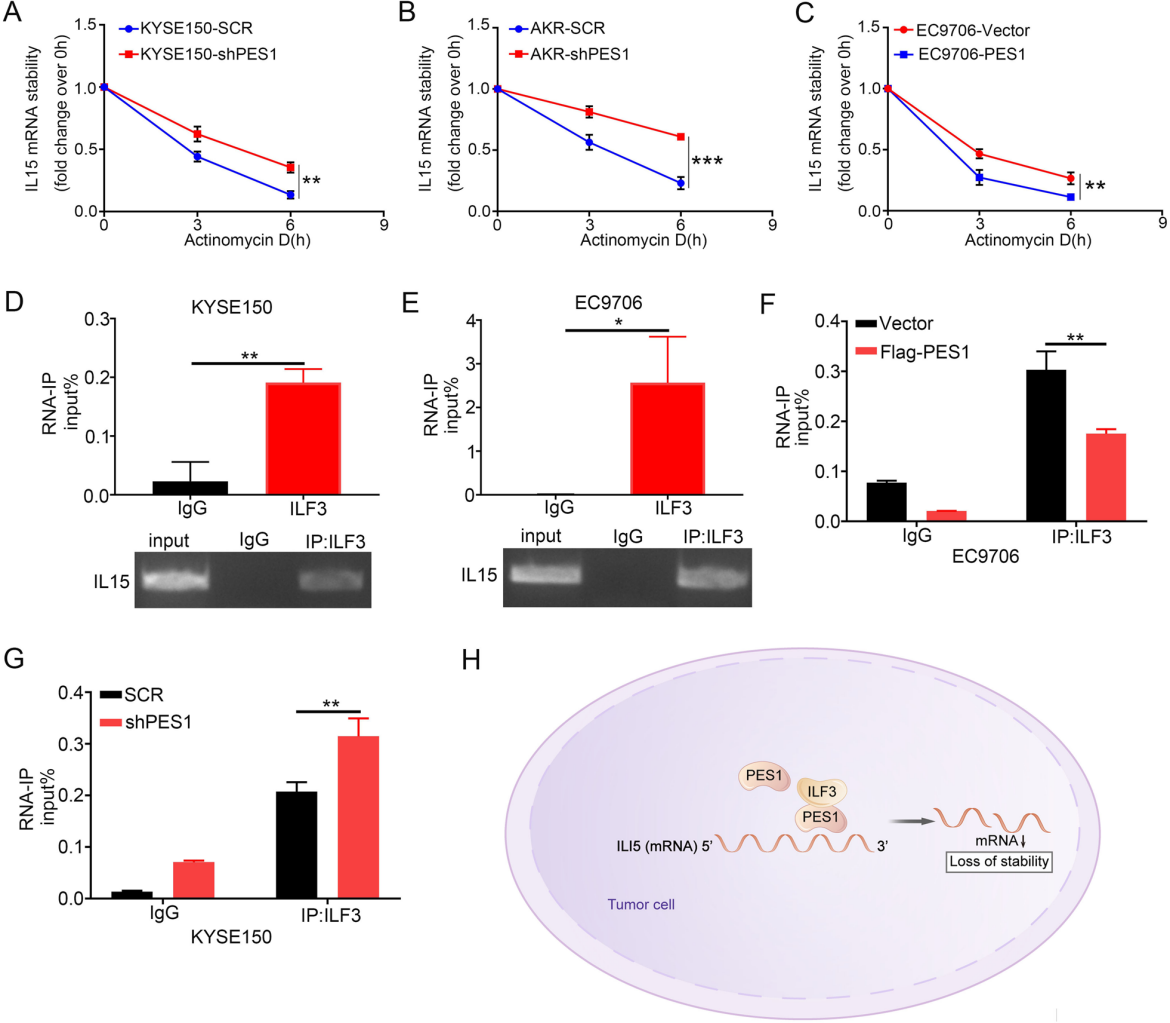
PES1 disrupts the interaction between ILF3 and IL15 mRNA. **A–C** IL15 mRNA stability was determined in PES1-knockdown KYSE150 cells (**A**) and AKR cells **B** and PES1-overexpressing EC9706 cells **C** treated with the transcription inhibitor actinomycin D for the indicated times (n = 3 independent experiments). **p < 0.01; ***p < 0.001. (D-E) RNA immunoprecipitation analysis of the binding of endogenous ILF3 and IL15 mRNA by ILF3 in KYSE150 (**D**) and EC9706 **E** cells (n = 3 independent experiments). **F**–**G** PES1 inhibits ILF3-IL15 mRNA interaction in EC9706 (**F**) and KYSE150 (**G**) cells by RNA immunoprecipitation with ILF3 antibody (n = 3 independent experiments). **p < 0.01; ***p < 0.001. **H** Working model showing that PES1 interferences the interaction between ILF3 and IL15 mRNA

We explored the mechanism by which the interaction between PES1 and ILF3 regulates the expression of *IL15*. Since the expression of PES1 affects *IL15* mRNA levels, and ILF3 is an RNA-binding protein (RBP) that stabilizes various transcripts [47], we hypothesized that PES1 might attenuate the stability of IL15 mRNA by competitive interaction with ILF3. To examine this hypothesis, we used actinomycin D to inhibit gene transcription and qRT-PCR was performed to measure the decay rate of *IL15*. As expected, the stability of IL15 mRNA was higher in shPES1 transfected KYSE150 and AKR cells than in their SCR counterparts (Fig. 6A, B). On the contrary, forced expression of PES1 in EC9706 cells obviously reduced the stability of IL15 mRNA (Fig. 6C). The RNA-IP assay confirmed the interaction between ILF3 and IL15 mRNA in extracts from KYSE150 and EC9706 cells (Fig. 6D, E). In addition, the interaction between ILF3 and IL15 mRNA was markedly attenuated by forced expression of PES1 (Fig. 6F) but enhanced by PES1 knockdown (Fig. 6G). Therefore, the results demonstrated that PES1 inhibited IL15 expression by disrupting the interaction between ILF3 and IL15 mRNA and ultimately promoted the degradation of IL15 mRNA (Fig. 6H).

### Targeting PES1 sensitized ESCC to ICB therapy

**Fig. 7:**
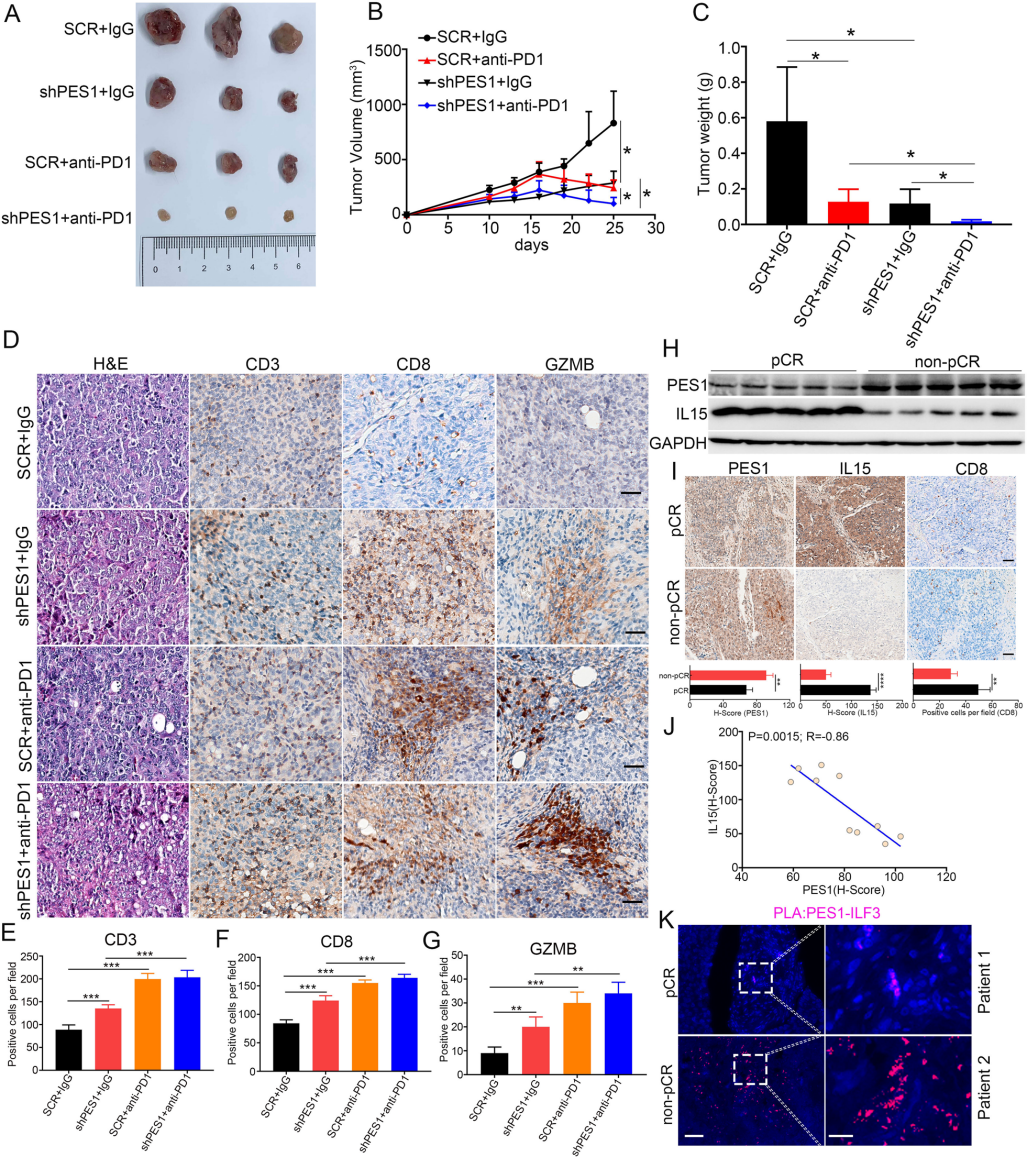
Knockdown PES1 sensitizes cancer cells to anti-PD1 therapy. **A** The representative image of subcutaneous tumors in indicated treatments. **B**, **C** Tumor volume over time **B** (Additional file 5: Dataset S4) and tumor weight **C** for AKR tumor-bearing mice (n = 4 mice per group). *p < 0.05. **D** HE and IHC were performed to examine the tumor area and detect the expression of CD3, CD8, and GZMB (Additional file 5: Dataset S4). (E–G) The CD3 + T cells (E), CD8 + T cells (**F**), GZMB + CD8 + T cells **G** were quantified (n = 4). Data shown as mean ± SD, statistical analysis by t test: *p < 0.05; **p < 0.01; ***p < 0.001; ****p < 0.0001. **H** Western blotting exhibits the expression of PES1 and IL15 from pre-treatment tumors of pCR and non-pCR patients with ESCC. pCR: pathological complete response; non-pCR: non-pathological complete response. **I** The representative IHC images of PES1, IL15 and CD8 from pre-treatment tumors of pCR (n = 5) and non-pCR (n = 5) patients and were quantified. Scale bar = 100 μm. *p < 0.05; **p < 0.01; ***p < 0.001; ****p < 0.0001. **J** Representative PLA images detecting the interaction between endogenous PES1 and ILF3 in pre-treatment pCR and non-pCR tissues. Scale bar = 100 μm

To explore the therapeutic significance of PES1 in ICB therapy in vivo, we inoculated SCR and shPES1 AKR cells into C57BL/6 mice, and anti-PD-1 antibody (*InVivo*MAb anti-mouse PD-1 (CD279), BE0273, BioXcell) was injected on days 7, 10 and 13 after tumor cell inoculation. The results indicated that the combination of anti-PD-1 antibody treatment and PES1 knockdown showed more potent suppressive effects on the subcutaneous growth of ESCC cells than either anti-PD-1 antibody treatment or PES1 knockdown alone (Fig. 7A–C). Moreover, the combination strategy significantly promoted the infiltration of CD8^+^ CTL compared with shPES1 and anti-PD-1 administration alone and increased the percentage of GZMB^+^/CD8^+^ CTL in the tumor microenvironment (Fig. 7D–G). To validate the clinical impact of PES1 in ESCC patients receiving ICB therapy, we collected the samples from ESCC patients receiving anti-PD-1 (Camrelizumab from Jiangsu Hengrui Pharmaceuticals Co., Ltd, Jiangsu, China) treatment in Department of Thoracic Surgery, Shanghai Chest Hospital, Shanghai, China. As shown in Fig. 7H, PES1 protein level were higher but IL15 protein level were lower in ESCC tissues from 5 non-pathological complete response (non-pCR) patients than in samples from five pathological complete response (pCR) patients. IHC results showed that the number of infiltrated CD8^+^ CTL were higher in pCR patient compared with non-pCR patient (Fig. 7I). Moreover, the expression level of PES1 and IL15 is not only related to the efficacy of immunotherapy, but also inverse correlation between protein levels of PES1 and IL15 (Fig. 7I, J). In addition, PLA suggested that the interaction between PES1 and ILF3 was attenuated in pCR patients compared to that in non-pCR patients (Fig. 7K and Additional file 1: Fig. S7). Therefore, the results confirmed that the efficacy of anti-PD-1 therapy was more potent in ESCC patients with low PES1 expression.

## Discussion

PES1 has been shown to be highly expressed in many cancer types and promote malignant behavior of tumor cells by affecting various oncogenic signaling pathways [48–51]. However, the function of PES1 in ESCC and the effect of PES1 on the immune landscape of ESCC remained largely unknown. Our study herein is the first report, according to the literature data mining, on the roles and molecular mechanisms of PES1 in the progression and ICB therapy of ESCC. Our findings suggest that PES1 promotes the tumor growth through inhibiting CD8^+^ CTL infiltration and inhibiting PES1 expression improves the efficacy of ICB therapy. Therefore, our study reveals novel physiological roles of PES1 and provides new insight on ICB therapy for ESCC.

Recently, ICB therapy has led to marked therapeutic responses in multiple malignancies including ESCC. However, most of patients do not achieve clinical benefits due to resistance with unclear mechanisms [52–54]. Therefore, precise markers should be identified to guide personalized immunotherapy. Recent researches have described tumor-infiltrating lymphocytes (TILs), especially CD8^+^ CTL, influencing clinical response and survival. High density of CD8^+^ CTL is dramatically correlated with more favorable overall survival and relapse-free survival. Clarifying and targeting the mechanisms utilized by tumors cells to prevent CD8^+^ CTL infiltration are critical to improve the efficacy of cancer immunotherapy in clinic. Several studies have demonstrated that tumor-intrinsic oncogenes can play key roles in regulating the immunosuppressive tumor microenvironment and tumor immune escape [55–58]. Successful identification of these genes would lead to new therapeutic strategies. Our online database analysis showed that the expression of PES1 in ESCC might be related to the infiltration of CD8^+^ CTL. Here, we reported that the functional role of PES1 in inhibiting CD8^+^ CTL infiltration into ESCC tissues. Mechanistic studies have demonstrated that PES1 interfered with the interaction between ILF3 and IL15 mRNA, reducing IL15 mRNA stability and gene expression. Knockdown of PES1 expression enhanced the effect of immunotherapy, indicating a potential therapeutic target for ESCC immunotherapy. Therefore, our work herein revealed a mechanism PES1 inhibits CD8^+^ CTL infiltration through the ILF3-IL15 axis, which in turn leads to tumor progression and dampening the antitumor immune response.

Cytokines play pivotal roles in modelling tumor microenvironment through regulating proliferation, differentiation, effector functions, and survival of immune cells. In recent years, some cytokines, including IL2, IL12, IL15, IL21, GM-CSF, and interferon-α, have been found to regulate the composition of tumor microenvironment and modulate the efficacy of immune therapy in murine cancer models [59, 60]. IL15 is critically required for the oncogene of NK cells and CD8^+^ CTL to induce proliferation, cytotoxic action, and infiltration of immune cells [61, 62]. In fact, many preclinical studies and clinical studies have demonstrated that IL15 has a synergistic effect on immune therapy in cancers [63–66]. However, short half-life and poor bioavailability of IL15 limit its therapeutic applications. Accordingly, improving the stability and minimizing the side effects of IL15 is urgent. It is also important to delineate the mechanisms of IL15 production in tumor cells and the upstream regulatory factors of IL15 might be potential pharmaceutical target. In this work, we demonstrated that PES1 could regulate the mRNA expression of IL15. Anti-IL15 treatment experiments demonstrated that PES1 inhibited CD8^+^ CTL infiltration through IL15. Further mechanistic studies revealed that PES1 competitively interacted with the RNA-binding protein ILF3 to interfere with ILF3-mediated stabilization of IL15 mRNA PES1 knockdown leaded to increased mRNA stability and protein level of IL15 as well as increased CD8^+^ CTL infiltration. ILF3, also known as NF90/NF110, encodes a double-stranded RNA-binding protein. ILF3 regulates the stability of various transcripts, such as IL2, Tau, VEGF, and MyoD [47, 67]. Interestingly, we found that ILF3 could interact with IL15 mRNA, and PES1 could interfere their interaction and affect the stability of IL15 mRNA. This finding allowed us to confirm that PES1 attenuates IL15 expression via ILF3-IL15 axis, thereby inhibiting CD8^+^ CTL infiltration and promoting tumor progression.

## Conclusions

**Fig. 8:**
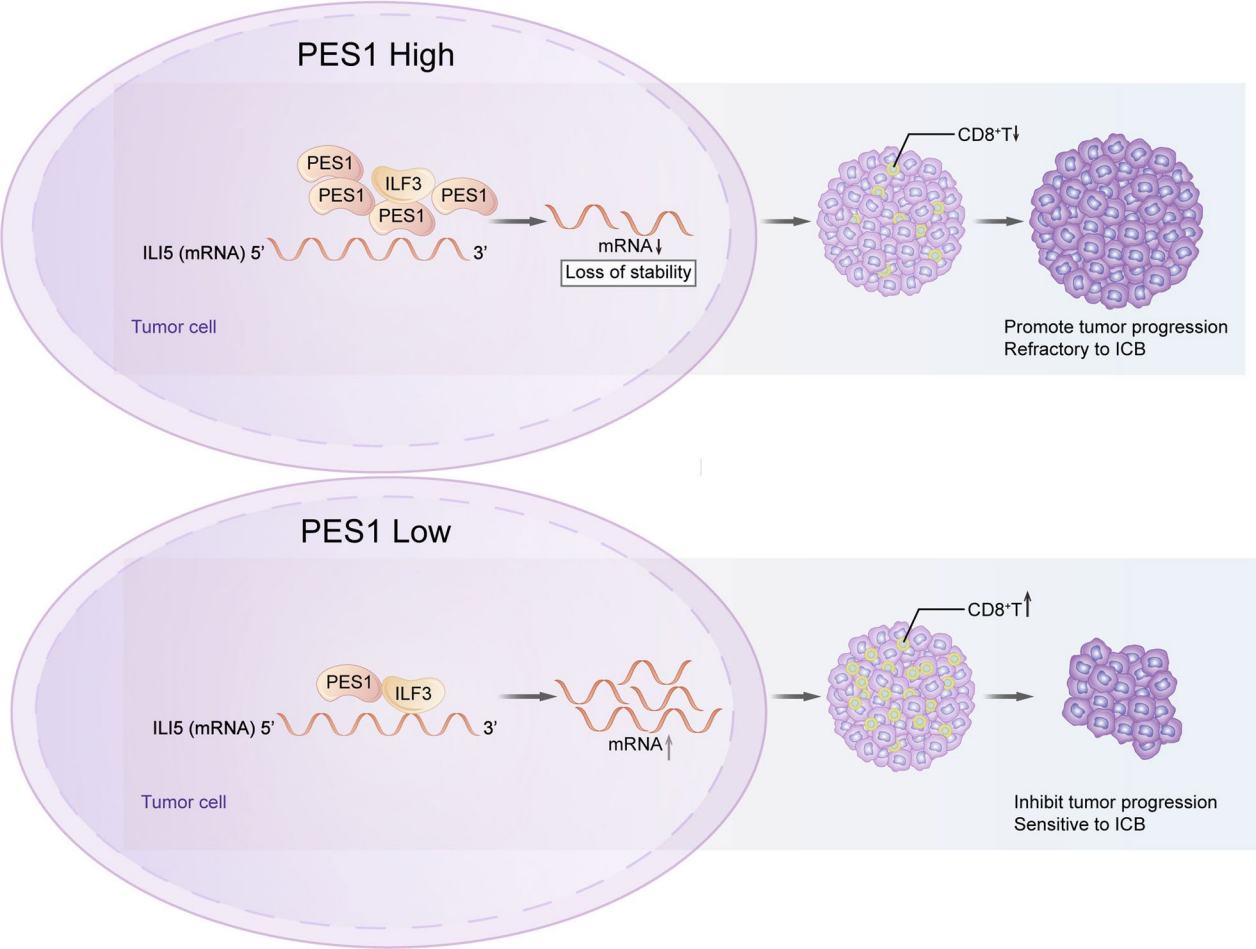
Schematic diagram of the regulation of CD8^+^ T cell infiltration by PES1. PES1 inhibits CD8^+^ CTL infiltration through disrupting the interaction between ILF3 and IL15 mRNA, which in turn leads to tumor progression and dampening the antitumor immune response

Altogether, our results reveal a new mechanism on intrinsic oncogene-mediated remodeling of tumor microenvironment and demonstrate that PES1 facilitate ESCC escape from immunosurveillance by interfering with the ILF3-IL15 axis in ESCC (Fig. 8). Dysregulation of PES1 is a previously unappreciated immune evasion mechanism in ESCC. In addition, our work provides a rationale for targeting PES1 to enhance immunotherapy effects on ESCC, indicating PES1 as a new therapeutic target to combine ICB therapy for patients with ESCC.

## Supplementary Information

**Additional file 1: Fig. S1.** Expression and clinical significance. **Fig. S2. **Effect of PES1 on tumor formation in vitro and in nude mice. **Fig. S3.** The infiltration analysis of other immune cells. **Fig. S4.** Correlation of PES1 and IL15 in ESCC. **Fig. S5.** Co-immunoprecipitation assay of PES1 with ILF3 and ILF2 in EC9706 cells. **Fig. S6.** The effect of PES1 and PES1 mut on the ILF3-dependent expression of IL15. **Fig. S7.** Representative PLA images detecting the interaction between endogenous PES1 and ILF3 in pre-treatment pCR and non-pCR tissues. **Table S1.** Consistently upregulated genes through Venn diagram analysis. **Table S2.** Pearson correlation analysis of CD8A with listed genes. **Table S3.** Pearson correlation analysis of CD8B with listed genes. **Table S4.** The correlation between PES1 expression levels and clinicopathologic features of the ESCC patients. **Table S5.** Primary antibodies used in this study.

**Additional file 2. **Additional dataset S1.

**Additional file 3.** Additional dataset S2.

**Additional file 4. **Additional dataset S3.

**Additional file 5. **Additional dataset S4.

## Data Availability

All data supporting the findings of this study are provided within the paper and its supplementary information. All additional information will be made available upon reasonable request from the authors.

## Abbreviations

ESCC: Esophageal squamous cell carcinoma
PES1: Pescadillo ribosomal biogenesis factor 1
IL15: Interleukin 15
ILF3: Interleukin enhancer binding factor 3
CTL: CD8^+^ cytotoxic T lymphocytes
ICB: Immune checkpoint blockade
PLA: Proximity ligation assay

## References

[1] Wei W, Zeng H, Zheng R, Zhang S, An L, Chen R, Wang S, Sun K, Matsuda T, Bray F (2020). Cancer registration in China and its role in cancer prevention and control. Lancet Oncol.

[2] Yang S, Lin S, Li N, Deng Y, Wang M, Xiang D, Xiang G, Wang S, Ye X, Zheng Y (2020). Burden, trends, and risk factors of esophageal cancer in China from 1990 to 2017: an up-to-date overview and comparison with those in Japan and South Korea. J Hematol Oncol.

[3] Kakeji Y, Oshikiri T, Takiguchi G, Kanaji S, Matsuda T, Nakamura T, Suzuki S (2021). Multimodality approaches to control esophageal cancer: development of chemoradiotherapy, chemotherapy, and immunotherapy. Esophagus.

[4] Petrasch S, Welt A, Reinacher A, Graeven U, König M, Schmiegel W (1998). Chemotherapy with cisplatin and paclitaxel in patients with locally advanced, recurrent or metastatic oesophageal cancer. Br J Cancer.

[5] Sun S, Yu H, Wang H, Zhang H, Wu X, Wang J, Chang J (2019). Phase II Study of S-1 plus Cisplatin as First-Line Therapy in Patients with Metastatic Esophageal Carcinoma. Oncology research and treatment.

[6] Zhang X, Shen L, Li J, Li Y, Li J, Jin M (2008). A phase II trial of paclitaxel and cisplatin in patients with advanced squamous-cell carcinoma of the esophagus. Am J Clin Oncol.

[7] Darvin P, Toor SM, Sasidharan Nair V, Elkord E (2018). Immune checkpoint inhibitors: recent progress and potential biomarkers. Exp Mol Med.

[8] Hodi FS, O'Day SJ, McDermott DF, Weber RW, Sosman JA, Haanen JB, Gonzalez R, Robert C, Schadendorf D, Hassel JC (2010). Improved survival with ipilimumab in patients with metastatic melanoma. N Engl J Med.

[9] Borghaei H, Paz-Ares L, Horn L, Spigel DR, Steins M, Ready NE, Chow LQ, Vokes EE, Felip E, Holgado E (2015). Nivolumab versus Docetaxel in Advanced Nonsquamous Non-Small-Cell Lung Cancer. N Engl J Med.

[10] Garon EB, Rizvi NA, Hui R, Leighl N, Balmanoukian AS, Eder JP, Patnaik A, Aggarwal C, Gubens M, Horn L (2015). Pembrolizumab for the treatment of non-small-cell lung cancer. N Engl J Med.

[11] Larkin J, Chiarion-Sileni V, Gonzalez R, Grob JJ, Cowey CL, Lao CD, Schadendorf D, Dummer R, Smylie M, Rutkowski P (2015). Combined Nivolumab and Ipilimumab or Monotherapy in Untreated Melanoma. N Engl J Med.

[12] Lu Z, Wang J, Shu Y, Liu L, Kong L, Yang L, Wang B, Sun G, Ji Y, Cao G (2022). Sintilimab versus placebo in combination with chemotherapy as first line treatment for locally advanced or metastatic oesophageal squamous cell carcinoma (ORIENT-15): multicentre, randomised, double blind, phase 3 trial. BMJ.

[13] Sun JM, Shen L, Shah MA, Enzinger P, Adenis A, Doi T, Kojima T, Metges JP, Li Z, Kim SB *et al*: Pembrolizumab plus chemotherapy versus chemotherapy alone for first-line treatment of advanced oesophageal cancer (KEYNOTE-590): a randomised, placebo-controlled, phase 3 study. *Lancet* 2021, 398(10302):759–771. 10.1016/S0140-6736(21)01234-4 34454674

[14] Wang ZX, Cui C, Yao J, Zhang Y, Li M, Feng J, Yang S, Fan Y, Shi J, Zhang X (2022). Toripalimab plus chemotherapy in treatment-naïve, advanced esophageal squamous cell carcinoma (JUPITER-06): A multi-center phase 3 trial. Cancer Cell.

[15] Kelly RJ, Ajani JA, Kuzdzal J, Zander T, Van Cutsem E, Piessen G, Mendez G, Feliciano J, Motoyama S, Lièvre A (2021). Adjuvant Nivolumab in Resected Esophageal or Gastroesophageal Junction Cancer. N Engl J Med.

[16] Li C, Zhao S, Zheng Y, Han Y, Chen X, Cheng Z, Wu Y, Feng X, Qi W, Chen K (2021). Preoperative pembrolizumab combined with chemoradiotherapy for oesophageal squamous cell carcinoma (PALACE-1). Eur J Cancer.

[17] Liu J, Li J, Lin W, Shao D, Depypere L, Zhang Z, Li Z, Cui F, Du Z, Zeng Y (2022). Neoadjuvant camrelizumab plus chemotherapy for resectable, locally advanced esophageal squamous cell carcinoma (NIC-ESCC2019): A multicenter, phase 2 study. Int J Cancer.

[18] Sihag S, Ku GY, Tan KS, Nussenzweig S, Wu A, Janjigian YY, Jones DR, Molena D (2021). Safety and feasibility of esophagectomy following combined immunotherapy and chemoradiotherapy for esophageal cancer. J Thorac Cardiovasc Surg.

[19] Liu J, Yang Y, Liu Z, Fu X, Cai X, Li H, Zhu L, Shen Y, Zhang H, Sun Y *et al*: Multicenter, single-arm, phase II trial of camrelizumab and chemotherapy as neoadjuvant treatment for locally advanced esophageal squamous cell carcinoma. *J Immunother Cancer* 2022, 10(3). 10.1136/jitc-2021-004291 PMC8961177 35338088

[20] Duan J, Xie Y, Qu L, Wang L, Zhou S, Wang Y, Fan Z, Yang S, Jiao S (2018). A nomogram-based immunoprofile predicts overall survival for previously untreated patients with esophageal squamous cell carcinoma after esophagectomy. J Immunother Cancer.

[21] Mlecnik B, Bindea G, Angell HK, Maby P, Angelova M, Tougeron D, Church SE, Lafontaine L, Fischer M, Fredriksen T (2016). Integrative Analyses of Colorectal Cancer Show Immunoscore Is a Stronger Predictor of Patient Survival Than Microsatellite Instability. Immunity.

[22] Hossain MA, Liu G, Dai B, Si Y, Yang Q, Wazir J, Birnbaumer L, Yang Y (2021). Reinvigorating exhausted CD8(+) cytotoxic T lymphocytes in the tumor microenvironment and current strategies in cancer immunotherapy. Med Res Rev.

[23] Raskov H, Orhan A, Christensen JP, Gögenur I (2021). Cytotoxic CD8(+) T cells in cancer and cancer immunotherapy. Br J Cancer.

[24] Baba Y, Nomoto D, Okadome K, Ishimoto T, Iwatsuki M, Miyamoto Y, Yoshida N, Baba H (2020). Tumor immune microenvironment and immune checkpoint inhibitors in esophageal squamous cell carcinoma. Cancer Sci.

[25] Baba Y, Yagi T, Kosumi K, Okadome K, Nomoto D, Eto K, Hiyoshi Y, Nagai Y, Ishimoto T, Iwatsuki M (2019). Morphological lymphocytic reaction, patient prognosis and PD-1 expression after surgical resection for oesophageal cancer. Br J Surg.

[26] Theivanthiran B, Haykal T, Cao L, Holtzhausen A, Plebanek M, DeVito NC, Hanks BA: Overcoming Immunotherapy Resistance by Targeting the Tumor-Intrinsic NLRP3-HSP70 Signaling Axis. *Cancers (Basel)* 2021, 13(19). 10.3390/cancers13194753 PMC8507548 34638239

[27] Xiong D, Wang Y, You M (2019). Tumor intrinsic immunity related proteins may be novel tumor suppressors in some types of cancer. Sci Rep.

[28] Takahashi M, Lio CJ, Campeau A, Steger M, Ay F, Mann M, Gonzalez DJ, Jain M, Sharma S (2021). The tumor suppressor kinase DAPK3 drives tumor-intrinsic immunity through the STING-IFN-β pathway. Nat Immunol.

[29] Sivaram N, McLaughlin PA, Han HV, Petrenko O, Jiang YP, Ballou LM, Pham K, Liu C, van der Velden AW, Lin RZ (2019). Tumor-intrinsic PIK3CA represses tumor immunogenecity in a model of pancreatic cancer. J Clin Invest.

[30] Cheng L, Yuan B, Ying S, Niu C, Mai H, Guan X, Yang X, Teng Y, Lin J, Huang J *et al*: PES1 is a critical component of telomerase assembly and regulates cellular senescence. *Sci Adv* 2019, 5(5):eaav1090. 10.1126/sciadv.aav1090 PMC6520020 31106266

[31] Jin X, Fang R, Fan P, Zeng L, Zhang B, Lu X, Liu T (2019). PES1 promotes BET inhibitors resistance and cells proliferation through increasing c-Myc expression in pancreatic cancer. J Exp Clin Cancer Res.

[32] Li J, Yu L, Zhang H, Wu J, Yuan J, Li X, Li M (2009). Down-regulation of pescadillo inhibits proliferation and tumorigenicity of breast cancer cells. Cancer Sci.

[33] Wang J, Sun J, Zhang N, Yang R, Li H, Zhang Y, Chen K, Kong D (2019). PES1 enhances proliferation and tumorigenesis in hepatocellular carcinoma via the PI3K/AKT pathway. Life Sci.

[34] Karakasheva TA, Waldron TJ, Eruslanov E, Kim SB, Lee JS, O'Brien S, Hicks PD, Basu D, Singhal S, Malavasi F (2015). CD38-Expressing Myeloid-Derived Suppressor Cells Promote Tumor Growth in a Murine Model of Esophageal Cancer. Cancer Res.

[35] Opitz OG, Harada H, Suliman Y, Rhoades B, Sharpless NE, Kent R, Kopelovich L, Nakagawa H, Rustgi AK (2002). A mouse model of human oral-esophageal cancer. J Clin Investig.

[36] Fan T, Chen J, Zhang L, Gao P, Hui Y, Xu P, Zhang X, Liu H (2016). Bit1 knockdown contributes to growth suppression as well as the decreases of migration and invasion abilities in esophageal squamous cell carcinoma via suppressing FAK-paxillin pathway. Mol Cancer.

[37] Hou G, Zhang Q, Wang L, Liu M, Wang J, Xue L (2010). mTOR inhibitor rapamycin alone or combined with cisplatin inhibits growth of esophageal squamous cell carcinoma in nude mice. Cancer Lett.

[38] Wang H, Qi Y, Lan Z, Liu Q, Xu J, Zhu M, Yang T, Shi R, Gao S, Liang G: Exosomal PD-L1 confers chemoresistance and promotes tumorigenic properties in esophageal cancer cells via upregulating STAT3/miR-21. *Gene Ther* 2022. 10.1038/s41434-022-00331-8 35440807

[39] Yang M, Liu R, Li X, Liao J, Pu Y, Pan E, Wang Y, Yin L (2015). Epigenetic Repression of miR-218 Promotes Esophageal Carcinogenesis by Targeting ROBO1. Int J Mol Sci.

[40] Li Y, Yang B, Ma Y, Peng X, Wang Z, Sheng B, Wei Z, Cui Y, Liu Z (2021). Phosphoproteomics reveals therapeutic targets of esophageal squamous cell carcinoma. Signal Transduct Target Ther.

[41] Liu W, Xie L, He YH, Wu ZY, Liu LX, Bai XF, Deng DX, Xu XE, Liao LD, Lin W (2021). Large-scale and high-resolution mass spectrometry-based proteomics profiling defines molecular subtypes of esophageal cancer for therapeutic targeting. Nat Commun.

[42] Kurz E, Hirsch CA, Dalton T, Shadaloey SA, Khodadadi-Jamayran A, Miller G, Pareek S, Rajaei H, Mohindroo C, Baydogan S *et al*: Exercise-induced engagement of the IL-15/IL-15Ralpha axis promotes anti-tumor immunity in pancreatic cancer. *Cancer Cell* 2022, 40(7):720–737 e725. 10.1016/j.ccell.2022.05.006 PMC9280705 35660135

[43] Rohrmoser M, Hölzel M, Grimm T, Malamoussi A, Harasim T, Orban M, Pfisterer I, Gruber-Eber A, Kremmer E, Eick D (2007). Interdependence of Pes1, Bop1, and WDR12 controls nucleolar localization and assembly of the PeBoW complex required for maturation of the 60S ribosomal subunit. Mol Cell Biol.

[44] Vumbaca F, Phoenix KN, Rodriguez-Pinto D, Han DK, Claffey KP (2008). Double-stranded RNA-binding protein regulates vascular endothelial growth factor mRNA stability, translation, and breast cancer angiogenesis. Mol Cell Biol.

[45] Zhu P, Jiang W, Cao L, Yu W, Pei Y, Yang X, Wan B, Liu JO, Yi Q, Yu L (2010). IL-2 mRNA stabilization upon PMA stimulation is dependent on NF90-Ser647 phosphorylation by protein kinase CbetaI. J Immunol.

[46] Soderberg O, Leuchowius KJ, Gullberg M, Jarvius M, Weibrecht I, Larsson LG, Landegren U (2008). Characterizing proteins and their interactions in cells and tissues using the in situ proximity ligation assay. Methods.

[47] Nourreddine S, Lavoie G, Paradis J, Ben El Kadhi K, Meant A, Aubert L, Grondin B, Gendron P, Chabot B, Bouvier M *et al*: NF45 and NF90 Regulate Mitotic Gene Expression by Competing with Staufen-Mediated mRNA Decay. *Cell Rep* 2020, 31(7):107660. 10.1016/j.celrep.2020.107660 32433969

[48] Fu Z, Jiao Y, Li YQ, Ke JJ, Xu YH, Jia BX, Liu B (2019). PES1 In Liver Cancer: A Prognostic Biomarker With Tumorigenic Roles. Cancer Manag Res.

[49] Wei S, Liu K, He Q, Gao Y, Shen L (2019). PES1 is regulated by CD44 in liver cancer stem cells via miR-105-5p. FEBS Lett.

[50] Cheng L, Li J, Han Y, Lin J, Niu C, Zhou Z, Yuan B, Huang K, Li J, Jiang K (2012). PES1 promotes breast cancer by differentially regulating ERα and ERβ. J Clin Invest.

[51] Li J, Zhou X, Lan X, Zeng G, Jiang X, Huang Z (2016). Repression of PES1 expression inhibits growth of gastric cancer. Tumour biology : the journal of the International Society for Oncodevelopmental Biology and Medicine.

[52] Zheng Y, Chen Z, Han Y, Han L, Zou X, Zhou B, Hu R, Hao J, Bai S, Xiao H (2020). Immune suppressive landscape in the human esophageal squamous cell carcinoma microenvironment. Nat Commun.

[53] Kudo T, Hamamoto Y, Kato K, Ura T, Kojima T, Tsushima T, Hironaka S, Hara H, Satoh T, Iwasa S (2017). Nivolumab treatment for oesophageal squamous-cell carcinoma: an open-label, multicentre, phase 2 trial. Lancet Oncol.

[54] Hirano H, Kato K (2019). Systemic treatment of advanced esophageal squamous cell carcinoma: chemotherapy, molecular-targeting therapy and immunotherapy. Jpn J Clin Oncol.

[55] Hamarsheh S, Groß O, Brummer T, Zeiser R (2020). Immune modulatory effects of oncogenic KRAS in cancer. Nat Commun.

[56] Zhao X, Liu S, Chen X, Zhao J, Li F, Zhao Q, Xie T, Huang L, Zhang Z, Qi Y (2021). L1CAM overexpression promotes tumor progression through recruitment of regulatory T cells in esophageal carcinoma. Cancer Biol Med.

[57] Li F, Kitajima S, Kohno S, Yoshida A, Tange S, Sasaki S, Okada N, Nishimoto Y, Muranaka H, Nagatani N (2019). Retinoblastoma Inactivation Induces a Protumoral Microenvironment via Enhanced CCL2 Secretion. Cancer Res.

[58] Koyama S, Akbay EA, Li YY, Aref AR, Skoulidis F, Herter-Sprie GS, Buczkowski KA, Liu Y, Awad MM, Denning WL (2016). STK11/LKB1 Deficiency Promotes Neutrophil Recruitment and Proinflammatory Cytokine Production to Suppress T-cell Activity in the Lung Tumor Microenvironment. Cancer Res.

[59] Bonati L, Tang L (2021). Cytokine engineering for targeted cancer immunotherapy. Curr Opin Chem Biol.

[60] Berraondo P, Sanmamed MF, Ochoa MC, Etxeberria I, Aznar MA, Pérez-Gracia JL, Rodríguez-Ruiz ME, Ponz-Sarvise M, Castañón E, Melero I (2019). Cytokines in clinical cancer immunotherapy. Br J Cancer.

[61] Bergamaschi C, Stravokefalou V, Stellas D, Karaliota S, Felber BK, Pavlakis GN: Heterodimeric IL-15 in Cancer Immunotherapy. *Cancers* 2021, 13(4). 10.3390/cancers13040837 PMC7922495 33671252

[62] Patidar M, Yadav N, Dalai SK (2016). Interleukin 15: A key cytokine for immunotherapy. Cytokine Growth Factor Rev.

[63] Rhode PR, Egan JO, Xu W, Hong H, Webb GM, Chen X, Liu B, Zhu X, Wen J, You L (2016). Comparison of the Superagonist Complex, ALT-803, to IL15 as Cancer Immunotherapeutics in Animal Models. Cancer Immunol Res.

[64] Ochoa MC, Minute L, López A, Pérez-Ruiz E, Gomar C, Vasquez M, Inoges S, Etxeberria I, Rodriguez I, Garasa S (2018). Enhancement of antibody-dependent cellular cytotoxicity of cetuximab by a chimeric protein encompassing interleukin-15. Oncoimmunology.

[65] Miller JS, Morishima C, McNeel DG, Patel MR, Kohrt HEK, Thompson JA, Sondel PM, Wakelee HA, Disis ML, Kaiser JC (2018). A First-in-Human Phase I Study of Subcutaneous Outpatient Recombinant Human IL15 (rhIL15) in Adults with Advanced Solid Tumors. Clin Cancer Res.

[66] Conlon KC, Lugli E, Welles HC, Rosenberg SA, Fojo AT, Morris JC, Fleisher TA, Dubois SP, Perera LP, Stewart DM (2015). Redistribution, hyperproliferation, activation of natural killer cells and CD8 T cells, and cytokine production during first-in-human clinical trial of recombinant human interleukin-15 in patients with cancer. Journal of clinical oncology : official journal of the American Society of Clinical Oncology.

[67] Castella S, Bernard R, Corno M, Fradin A, Larcher JC (2015). Ilf3 and NF90 functions in RNA biology. Wiley interdisciplinary reviews RNA.

