# Supplementary material for: PES1 reduces CD8+ T cell infiltration and immunotherapy sensitivity via interrupting ILF3-IL15 complex in esophageal squamous cell carcinoma
Source: J Biomed Sci. 2023 Mar 23;30:20. doi: 10.1186/s12929-023-00912-8 (PMC10037800; doi:10.1186/s12929-023-00912-8)
Supplement: Supplementary file 1 — Additional file 1: Fig. S1. Expression and clinical significance. Fig. S2. Effect of PES1 on tumor formation in vitro and in nude mice. Fig. S3. The infiltration analysis of other immune cells. Fig. S4. Correlation of PES1 and IL15 in ESCC. Fig. S5. Co-immunoprecipitation assay of PES1 with ILF3 and ILF2 in EC9706 cells. Fig. S6. The effect of PES1 and PES1 mut on the ILF3-dependent expression of IL15. Fig. S7. Representative PLA images detecting the interaction between endogenous PES1 and ILF3 in pre-treatment pCR and non-pCR tissues. Table S1. Consistently upregulated genes through Venn diagram analysis. Table S2. Pearson correlation analysis of CD8A with listed genes. Table S3. Pearson correlation analysis of CD8B with listed genes. Table S4. The correlation between PES1 expression levels and clinicopathologic features of the ESCC patients. Table S5. Primary antibodies used in this study. [file 12929_2023_912_MOESM1_ESM.docx]

**PES1 reduces CD8^+^ T cell infiltration and immunotherapy sensitivity via interrupting ILF3-IL15 complex in esophageal squamous cell carcinoma**

Ning Ma^1, #^, Rong Hua^1,#^, Yang Yang^1,#^ Zhi-Chao Liu^1^, Jie Pan^1^, Bo-Yao Yu^1^, Yi-Feng Sun^1^, Dong Xie^1,2^, Yan Wang^1,3,*^, Zhi-Gang Li^1,*^

^1^Department of Thoracic Surgery, Shanghai Chest Hospital, School of medicine, Shanghai Jiao Tong University, Shanghai, China.

^2^CAS Key Laboratory of Nutrition, Metabolism and Food Safety, Shanghai Institute of Nutrition and Health, University of Chinese Academy of Sciences, Chinese Academy of Sciences, Shanghai, China.

^3^Institute of Pathology and Southwest Cancer Center, Southwest Hospital, Army Medical University (Third Military Medical University), Chongqing, China.

^#^These authors contributed equally to this work.

*Correspondence: Yan Wang: [wang_yan1977@hotmail.com](mailto:wang_yan1977@hotmail.com)

Zhi-Gang Li: [zhigang.li@shsmu.edu.cn](mailto:zhigang.li@shsmu.edu.cn)


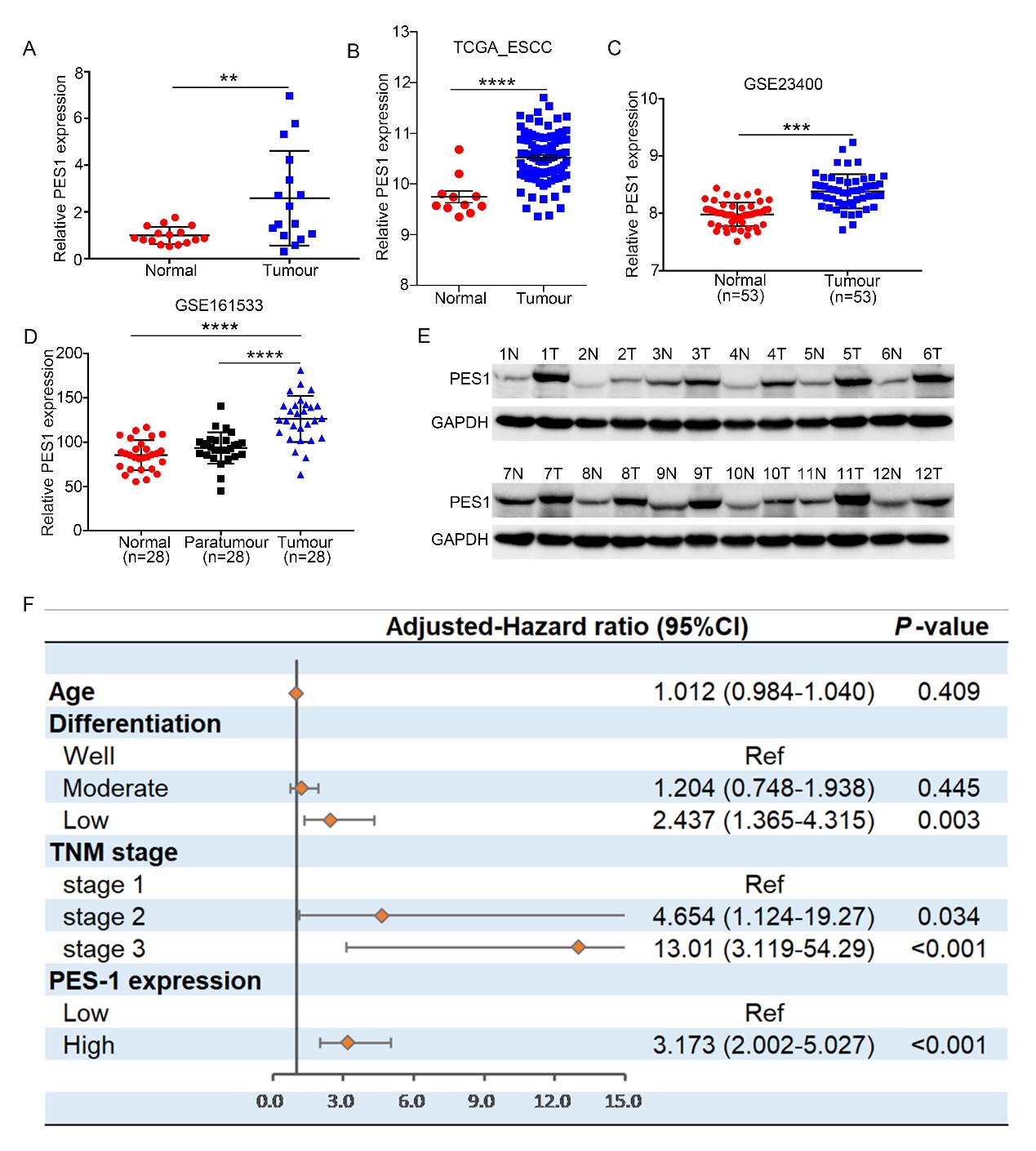


**Fig. S1. Expression and clinical significance of PES1 in ESCC.** (A) The mRNA level of PES1 in 16 ESCC tissues and 16 adjacent tissues was analyzed with a *t test*. **, *p*<0.01. (B-D) Dot plots comparing levels of PES1 mRNA in normal tissues (n = 11) and ESCC tissues (n = 96) in data sets from TCGA_ESCC (B), GSE23400 (C), and GSE161533 (D). (E) Western blotting was performed to detect the expression of PES1 in 12 ESCC tissues (T) and matched adjacent tissues (N). GAPDH is used as internal control. (F) Multivariate survival analysis of PES1 expression on overall survival in the ESCC cohort. The multivariate Cox-proportional hazards model was adjusted for age, differentiation, TNM stage and PES-1 expression.


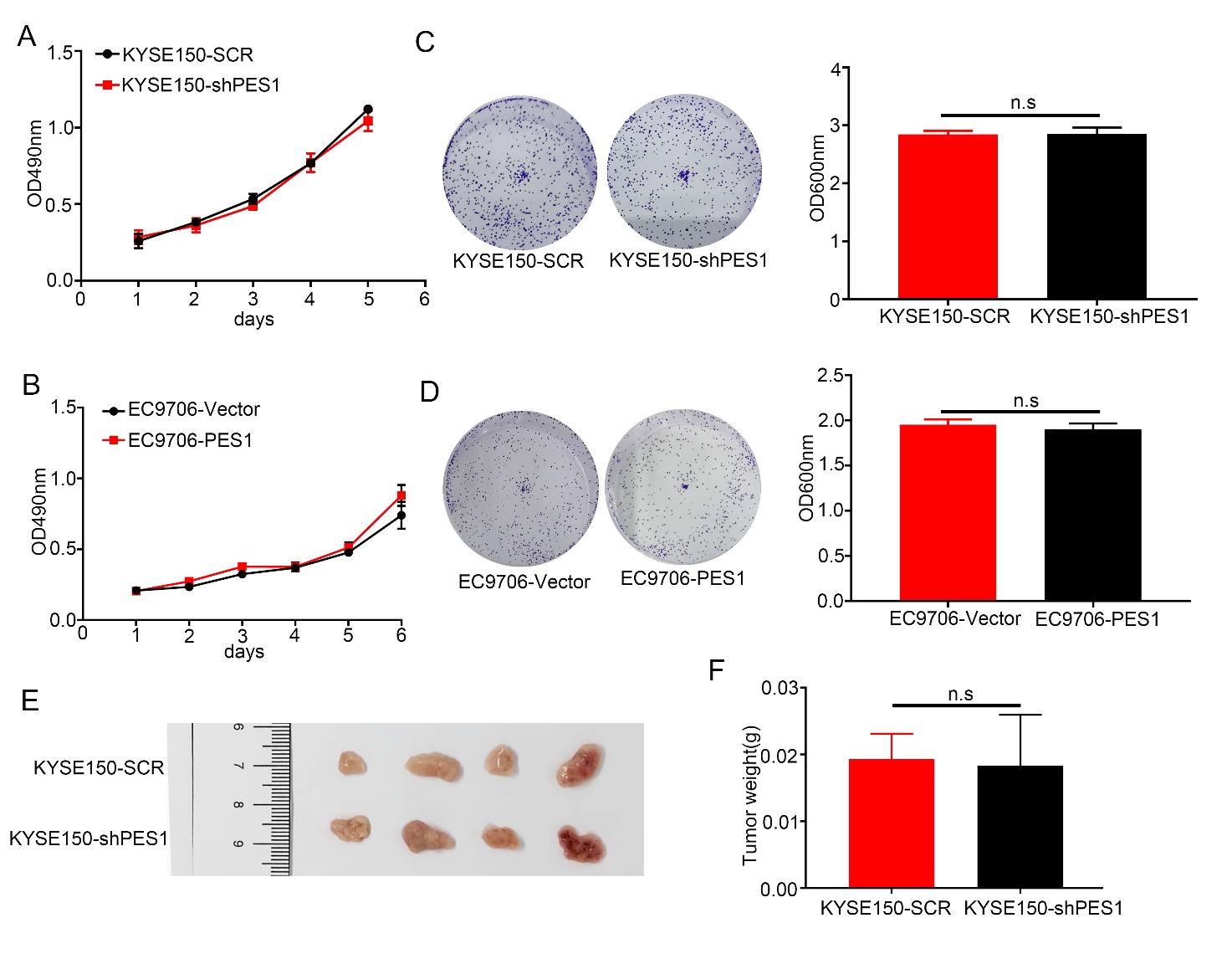


**Fig. S2. Effect of PES1 on tumor formation in vitro and in nude mice.** (A-B) MTT assay examining the effect of PES1 on the growth of KYSE150 and EC9706 cells. (C-D) The crystal violet assay examining the effect of PES1 on the growth of KYSE150 and EC9706 cells. (E-F) A tumororigenicity assay detect the effect of PES1 on tumor growth in nude mice (n=4 in each group). n.s: no significant difference.

**
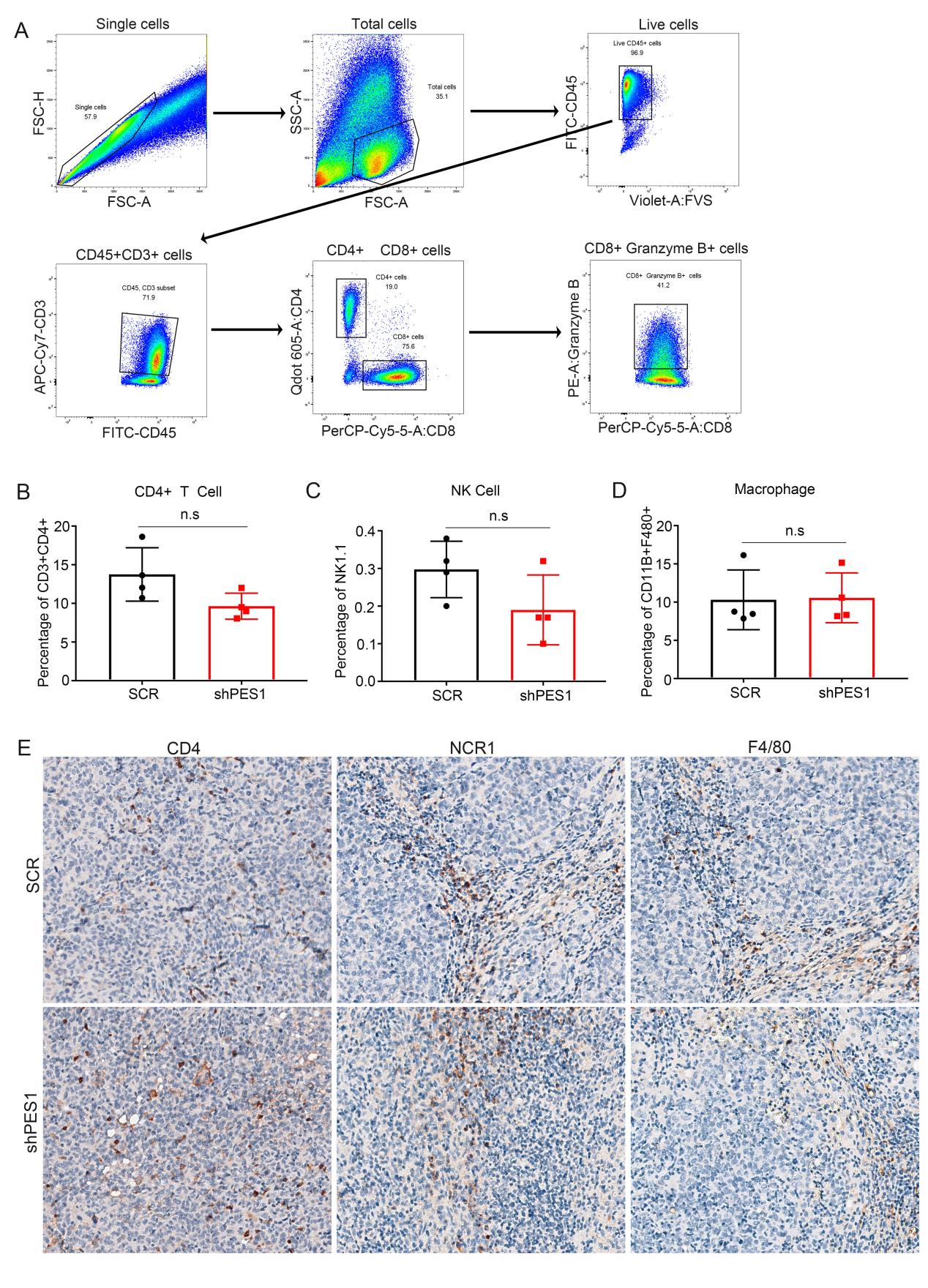
**

**Fig. S3. The infiltration analysis of other immune cells.** (A) Representative flow-cytometry gating strategy for quantifying the numbers of various immune effector cell subsets in murine tumour. (B-D) Effect of PES1 knockdown in AKR tumors on the other cells infiltration. Representative quantification of CD3^+^ CD4^+^ T cells (B), NK1.1 cells (C), and CD11B^+^F4/80^+^ cells (D) for the indicated groups (n = 4 mice per group). n.s: no significant difference. (E) IHC were performed to detect the expression of CD4, NCR1, and F4/80. Scale bar = 100 μm.

**
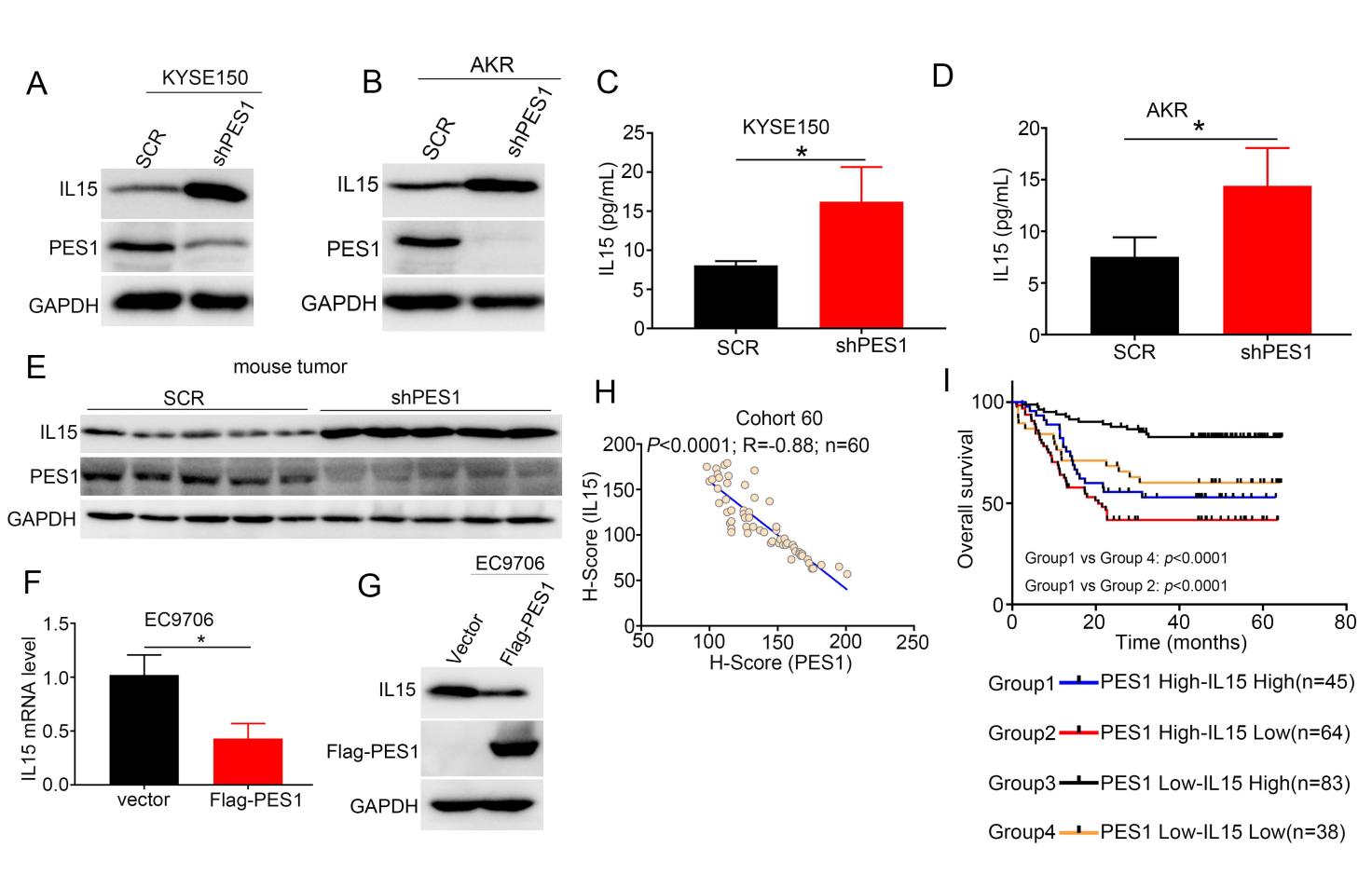
**

**Fig. S4. Correlation of PES1 and IL15 in ESCC.** (A-B) Western blot analysis of IL15 in PES1 knockdown ESCC cells, KYSE150 (A), AKR (B). (C-D) ELISA for analyzing the level of secreted IL15 from the culture medium in PES1 knockdown ESCC cells, KYSE150 (C), AKR (D). (E) Western blot analysis of IL15 expression in tumor xenografts. (F-G) qPCR and western blots shown the expression of IL15 in PES1-overexpressing EC9706 cells. **P* < 0.05. (H) Correlation between PES1 expression and IL15 expression in the 60 ESCC samples. Linear regression analysis showing the inverse correlation between PES1 and IL15. (I) The correlation of PES1/IL15 expression with the overall survival of patients with ESCC was analyzed.


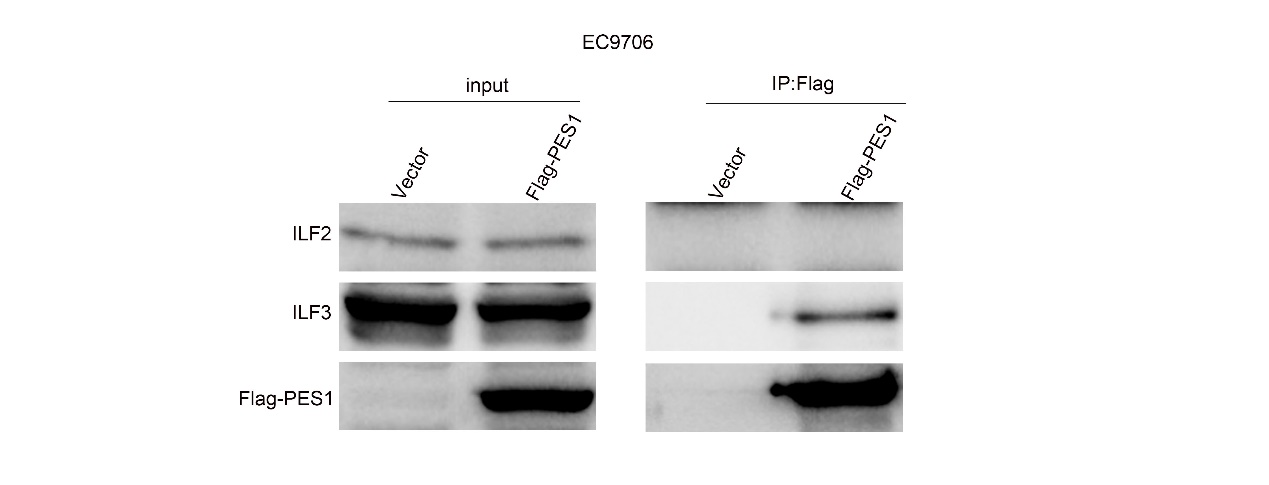


**Fig. S5.** Co-immunoprecipitation assay of PES1 with ILF3 and ILF2 in EC9706 cells.


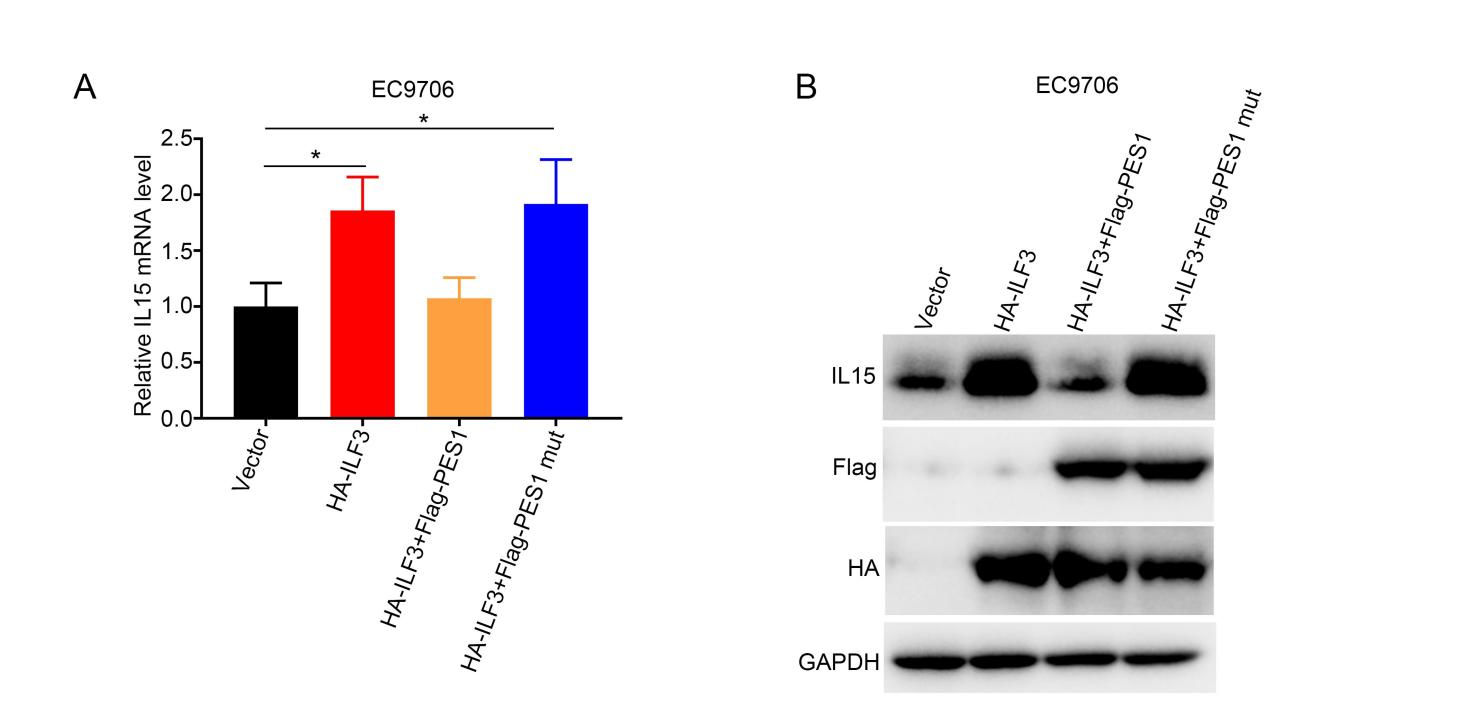


**Fig. S6.** **The effect of PES1 and PES1 mut on the ILF3-dependent expression of IL15.** qPCR (A) and western blotting (B) result for the expression of IL15 in different conditions. **P* < 0.05.


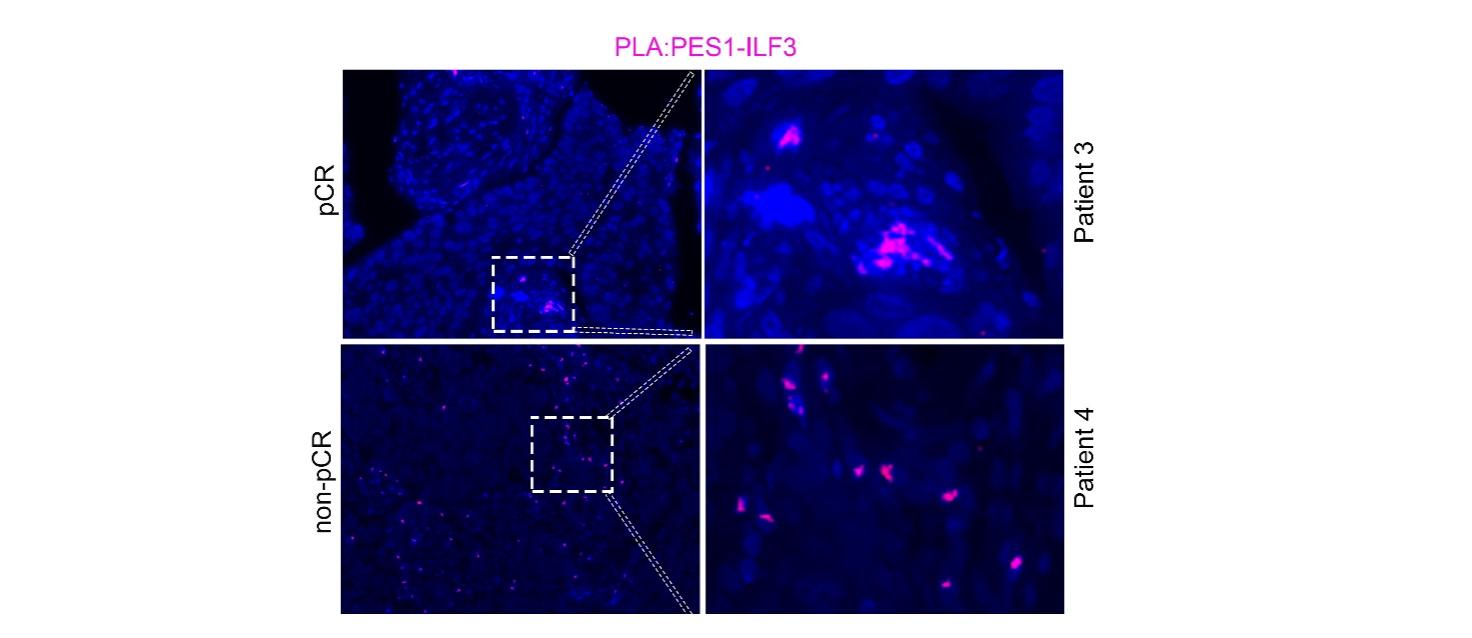


**Fig. S7.** Representative PLA images detecting the interaction between endogenous PES1 and ILF3 in pre-treatment pCR and non-pCR tissues. Scale bar = 100 μm.

**Table S1. Consistently upregulated genes through Venn diagram analysis.**

| **Protein (*Nat Commun.* 2021; 12: 4961; *P* < 0.001, FC > 1.4 from *Signal Transduct Target Ther.* 2021; 6: 381)** | **mRNA (*P* < 0.001, FC > 1.4 in GSE45670; *P* < 0.001, FC > 1.4 in GSE161533; *P* < 0.001, FC > 1.04 in GSE23400; *P* < 0.001, FC > 1.04 in TCGA_ESCC)** | **Protein and mRNA** |
| --- | --- | --- |
| RCN3; MMP2; HMCES; APOL2; MYO1B; PANX1; CYBA; PLAU; SUMO1; TPX2; IGF2BP3; SLC33A1; HMGB3; HLTF; ANP32E; FKBP7; SAAL1; BASP1; CGAS; NASP; ELANE; KPNA2; UAP1L1; ANLN; P3H1; PCYT1A; MSH6; SRSF6; CDK1; DTX3L; KIF11; NCF2; NOP2; TAP1; EPX; KIFC1; UBXN7; SLC1A5; UBE2L6; WDR12; MASTL; RUNX1; SMC4; PARP14; MNDA; SSR3; ATP6V1C1; PFN2; CKS1B; MCM3; LAMB3; SSRP1; CHAF1A; OAS3; LIMA1; MORF4L2; SUB1; SMARCA5; FKBP10; MPO; KIF4A; WARS; PCNA; TMEM189; DDX60; KYNU; SLC38A2; XAF1; CASP14; POMK; B2M; KIF2C; GFM1; GALNT2; LIPA; IL4I1; PYCR1; HTRA3; SFRP4; TYMS; CAMP; MCM5; MXRA5; CASP7; ITGB2; IL36G; CTSD; TFRC; TXNDC12; SUPT16H; GOPC; RFC4; PLOD3; PLOD2; SOAT1; PBK; AZU1; L1RE1; FUT8; NNMT; AMPD3; TRIP13; MMP1; STAT1; CRTAP; SLC2A1; EPCAM; PSTPIP2; ISG15; PES1; FXR1; MX2; DNAJB11; RNF213; PARP1; KIF23; NSUN2; ITGAM; NCAPH; TK1; PLBD2; EIF2AK2; SLC12A7; EPPK1; P4HA2; SMC2; IKBIP; TNC; PML; IGF2BP2; SERPINH1; FAP; PSAP; TPM4; PCLAF; TFB2M; KIDINS220; DNASE2; LOXL2; ATAD2; PHF6; DNMT1; POSTN; TACC3; MMP14; TAP2; UBE2C; FSCN1; MCM7; PRC1; SLC1A4; IFIT1; ARMC9; GBP1; OAS2; IFI30; ESF1; RRM2; TOP2A; FEN1; ICAM1; TNS4; SLC16A3; THBS2; CYBB; LTF; MYDGF; NCAPD2; MTHFD1L; NCL; MCM2; MCM4; HTRA1; DDX21; ADAR; MSH2; DLGAP5; HAT1; NMI; IFI35; P4HA1; VCAN; HERC5; MKI67; STAT2; RBMX; MX1; CBX3; SULF1; MBD3; MARCKSL1; LAMC2; NOLC1; MCM6; APOBEC3G; PPM1G; FZD6; CFDP1; FNDC3B; C5orf22; COLGALT1; PPP1R14C; FCER1G; SPARC; CLEC11A; GMPS; FKBP9; CTSZ; S100A12; GBP5; CTHRC1; UHRF1; PYCR2; CEBPB; BPI; FMNL2; PARP9; NPL; PLAUR; LPCAT1; LRPAP1; P3H3; SLC16A1; TYMP; VRK1; APP; IFI44; NUSAP1; IGF2BP1; MAGEA4; XPO5; IFIT3; ITPR3; MMP9; STEAP1; TOP1; PLOD1 | CDK1; KIF4A; MCM5; SUPT16H; PES1; KIF23; EIF2AK2; MMP14; PRC1; OAS2; MCM2; MCM4; ADAR; CBX3; TYMP; RFC4; TRIP13; ISG15; SMC2; UBE2C; RRM2; TOP2A; FEN1; FZD6; TPX2; IGF2BP3; NASP; KPNA2; LAMB3; SSRP1; CHAF1A; STAT1; SLC2A1; TACC3; FSCN1; MCM7; DLGAP5; HAT1; MKI67; MYO1B; HMGB3; CKS1B; OAS3; PCNA; KYNU; KIF2C; TFRC; PBK; NCAPH; ATAD2; DNMT1; IFI30; NMI; NOLC1; GMPS; NUSAP1; CD44; IRAK1; TMSB10; HIST1H4J; CCNB1; IPO9; BRCA1; CDC25B; RUNX3; LMNB2; COL1A1; CCNE1; FOXM1; USP14; TUBB; PLXNA1; FUS; RSRC1; DDX18; SNAPC1; ATR; LAGE3; COL7A1; TSN; PTPN2; KIF14; SMC6; MAD2L1; C1orf112; MRTO4; RNF7; SNRPC; NUP85; SQLE; MCM10; PSMD12; MEX3D; MELK; GEMIN6; HN1; TBCCD1; NDC80; OIP5; CCNA2; TMEM132A; CKS2; TDP1; DEPDC1; ANXA2; INTS7; NUTF2; TRMT1; SPAG5; BRCA2; XPO6; AGRN; MRPL13; CCNB2; CDT1; FCF1; LRP8; HOXC10; TBL1XR1; TMEM39B; FARSA; CCNE2; CDC45; CCT3; SLC3A2; TGS1; CDK2; JUP; CDC23; NPM3; MAGOH; EXO1; EBNA1BP2; CENPI; STX6; HOXB7; HNRNPU; PRCC; CCT6A; LAPTM4B; NEK2; SHMT2; METTL1; CDC25A; RAE1; BID; GTF2E1; SFPQ; NUP93; DDX27; TAGLN2; ITGB4; EZH2; CHEK1; NEIL3; SKP2; KIF18B; PPAP2C; TRAF3; SNRPF; LSG1; PUS1; ATP2C1; DBF4; KDM1A; EFNA1; BOLA2; NAA50; HOXA10; ABCE1; SPHK1; RUVBL1; FBXO5; CENPA; CSE1L; CENPN; CDC20; DKC1; GART; PLK4; H2AFX; BUB1; POLQ; HEATR1; DHX9; PRPF40A; KRT14; CLSTN1; RFWD3; UBE2S; MRPL3; IFI6; STIL; TFDP1; LSM5; PUS7; NCBP2; HPRT1; HELLS; FANCI; PSMA7; TUBG1; PSMB5; CXCL1; SHFM1; PKMYT1; KIF18A; TMX1; BAZ1A; ZFP64; NIT2; ERCC6L; CKAP5; PNO1; MORC2; DSN1; TDO2; DERL1; MAP4K4; ATP1B3; NUP62; CDKN3; E2F3; CENPM; GPC1; BTG3; PAK2; RBL1; FAM64A; CENPF; PFDN2; CDCA8; POLR2G; BYSL; ADAM12; G3BP1; NUP155; DDX11; HOXD11; GINS1; DSCC1; BIRC5; TRA2B; KNTC1; LRRC8D; IPO4; BOP1; LAMP3; RACGAP1; DNMT3B; PSMC3IP; PTGES; CDC6; SAC3D1; AURKA; DHFR; PRIM2; AURKB; IFI16; TIPIN; MRPS28; CTSB; UMPS; FANCA; RAD54B; RANBP1; TMEM185B; PRKDC; PSMD2; POP7; DNA2; SRRT; SNRPG; XPOT; ZWINT; TGFB1; BCL11B; HIST1H2AG; GTSE1; RCC1; PTTG1; BNC1; ECT2; XCL1; CDH3; GGH; EXT1; CENPE; PARP12; SFXN1; HNRNPA2B1; PPP1R14B; HOXD10; CEP55; DTYMK; RBBP8; BMP1; ARPC1B; MFAP2; CNIH4; NUP107; TIMELESS; RPA3; MAFB; ENO1; SCO2; EXOSC4; C16orf80; ADAM17; ADPGK; SHOX2; SREBF1; TLR2; LIMK1; KIAA0101; EPHB2; NME1; POLA2; PYCARD; GINS2; ARTN; TOPBP1; HOMER3; NETO2; FBXL6; EIF3B; ILF2; PSMA3; ACTL6A; PLK1; BRMS1; TNFSF10; ASF1B; TAF2; ZWILCH; MYBL2; CCDC109B; SNRPE; BLM; PSMB2; SNRPD1; NCAPG2; PTK7; IL1RAP; GABPB1; ILF3; TROAP; RAN; LSM7; HSP90AA1; MEN1; NIP7; SHCBP1; TAF1A; CBFB; DPP3; ASPM; RFC2; CDCA3; SLC7A5; POLE2; CAD; CKAP2; DNAJC2; UBAP2L; SPC25; WDHD1; KIF15; FLAD1; POLR2H; BUB1B; HJURP; TCF3; RNASEH2A; EIF2AK1; DTL; ALG3; RAD51; FERMT1; HMMR; GMNN; CCT5; KIF20A; SNRPB; F11R; LGALS8; DDR1; TTK; SLC38A7; NCAPG; BAX; TP63; FAM49B; SLC39A6; CTSC; | PES1; CDK1; KIF4A; MCM5; SUPT16H; KIF23; EIF2AK2; MMP14; PRC1; OAS2; MCM2; MCM4; ADAR; CBX3; TYMP; RFC4; TRIP13; ISG15; SMC2; UBE2C; RRM2; TOP2A; FEN1; FZD6; TPX2; IGF2BP3; NASP; KPNA2; LAMB3; SSRP1; CHAF1A; STAT1; SLC2A1; TACC3; FSCN1; MCM7; DLGAP5; HAT1; MKI67; MYO1B; HMGB3; CKS1B; OAS3; PCNA; KYNU; KIF2C; TFRC; PBK; NCAPH; ATAD2; DNMT1; IFI30; NMI; NOLC1; GMPS; NUSAP1 |

**Table S2. Pearson correlation analysis of CD8A with listed genes.**

| **Gene Symbol** | **Case Number** | **Pearson R** | **P value (two-tailed)** |
| --- | --- | --- | --- |
| **PES1** | **107** | **-0.3245** | **0.0006** |
| SLC2A1 | 107 | -0.2687 | 0.0051 |
| TFRC | 107 | -0.2623 | 0.0063 |
| MYO1B | 107 | -0.257 | 0.0075 |
| MMP14 | 107 | -0.2329 | 0.0158 |
| FSCN1 | 107 | -0.2038 | 0.0353 |
| IGF2BP3 | 107 | -0.1989 | 0.04 |
| KPNA2 | 107 | -0.1684 | 0.083 |
| HAT1 | 107 | -0.1656 | 0.0883 |
| RRM2 | 107 | -0.1629 | 0.0936 |
| MCM4 | 107 | -0.1588 | 0.1024 |
| SSRP1 | 107 | -0.1583 | 0.1035 |
| NCAPH | 107 | -0.1579 | 0.1043 |
| MCM2 | 107 | -0.152 | 0.1182 |
| PRC1 | 107 | -0.1476 | 0.1291 |
| KIF23 | 107 | -0.1398 | 0.151 |
| HMGB3 | 107 | -0.1364 | 0.1612 |
| GMPS | 107 | -0.1362 | 0.1617 |
| MCM5 | 107 | -0.1336 | 0.1701 |
| FZD6 | 107 | -0.1313 | 0.1776 |
| SUPT16H | 107 | -0.1311 | 0.1784 |
| KIF4A | 107 | -0.1244 | 0.2016 |
| EIF2AK2 | 107 | -0.1228 | 0.2078 |
| TRIP13 | 107 | -0.1219 | 0.2108 |
| SMC2 | 107 | -0.1064 | 0.2752 |
| FEN1 | 107 | -0.1057 | 0.2786 |
| DLGAP5 | 107 | -0.1033 | 0.2899 |
| RFC4 | 107 | -0.1022 | 0.2948 |
| LAMB3 | 107 | -0.1014 | 0.2987 |
| TPX2 | 107 | -0.1004 | 0.3033 |
| MKI67 | 107 | -0.08702 | 0.3728 |
| NUSAP1 | 107 | -0.08051 | 0.4097 |
| PBK | 107 | -0.07 | 0.4737 |
| DNMT1 | 107 | -0.06804 | 0.4862 |
| TOP2A | 107 | -0.05802 | 0.5528 |
| CBX3 | 107 | -0.05171 | 0.5969 |
| KIF2C | 107 | -0.04454 | 0.6488 |
| ATAD2 | 107 | -0.03769 | 0.7 |
| MCM7 | 107 | -0.03317 | 0.7345 |
| CDK1 | 107 | -0.03173 | 0.7456 |
| CKS1B | 107 | -0.02969 | 0.7614 |
| CHAF1A | 107 | -0.02445 | 0.8026 |
| NOLC1 | 107 | -0.02032 | 0.8354 |
| UBE2C | 107 | 0.01763 | 0.8569 |
| TACC3 | 107 | 0.02374 | 0.8083 |
| PCNA | 107 | 0.02899 | 0.7669 |
| ADAR | 107 | 0.05746 | 0.5566 |
| TYMP | 107 | 0.1386 | 0.1545 |
| OAS3 | 107 | 0.1617 | 0.096 |
| KYNU | 107 | 0.1631 | 0.0932 |
| NASP | 107 | 0.1745 | 0.0723 |
| ISG15 | 107 | 0.1793 | 0.0647 |
| OAS2 | 107 | 0.3079 | 0.0013 |
| NMI | 107 | 0.3919 | < 0.0001 |
| STAT1 | 107 | 0.4283 | < 0.0001 |
| IFI30 | 107 | 0.6172 | < 0.0001 |

**Remarks:** In Table S2, the case number 107 included 11 non-tumor esophageal tissues and 96 ESCC samples.

**Table S3. Pearson correlation analysis of CD8B with listed genes.**

| **Gene Symbol** | **Case Number** | **Pearson R** | **P value (two-tailed)** |
| --- | --- | --- | --- |
| **PES1** | **107** | **-0.359** | **0.0001** |
| TFRC | 107 | -0.322 | 0.0007 |
| SLC2A1 | 107 | -0.3039 | 0.0015 |
| MYO1B | 107 | -0.2974 | 0.0019 |
| KPNA2 | 107 | -0.2304 | 0.0169 |
| MMP14 | 107 | -0.2257 | 0.0194 |
| FSCN1 | 107 | -0.2066 | 0.0328 |
| SSRP1 | 107 | -0.2056 | 0.0336 |
| IGF2BP3 | 107 | -0.2042 | 0.0349 |
| MCM4 | 107 | -0.1962 | 0.0428 |
| FZD6 | 107 | -0.1943 | 0.0449 |
| PRC1 | 107 | -0.1868 | 0.0541 |
| HAT1 | 107 | -0.1839 | 0.058 |
| NCAPH | 107 | -0.1793 | 0.0645 |
| SMC2 | 107 | -0.1793 | 0.0646 |
| SUPT16H | 107 | -0.1752 | 0.0711 |
| RRM2 | 107 | -0.1706 | 0.0789 |
| GMPS | 107 | -0.1623 | 0.0948 |
| KIF23 | 107 | -0.1619 | 0.0958 |
| FEN1 | 107 | -0.1594 | 0.101 |
| MCM2 | 107 | -0.1559 | 0.1088 |
| EIF2AK2 | 107 | -0.1531 | 0.1153 |
| LAMB3 | 107 | -0.1464 | 0.1323 |
| KIF4A | 107 | -0.1461 | 0.1332 |
| MCM5 | 107 | -0.1362 | 0.1619 |
| HMGB3 | 107 | -0.1344 | 0.1675 |
| DLGAP5 | 107 | -0.1329 | 0.1722 |
| MKI67 | 107 | -0.1287 | 0.1863 |
| TRIP13 | 107 | -0.1194 | 0.2205 |
| TPX2 | 107 | -0.1116 | 0.2526 |
| RFC4 | 107 | -0.1104 | 0.2577 |
| DNMT1 | 107 | -0.1099 | 0.2597 |
| TOP2A | 107 | -0.0907 | 0.3528 |
| NUSAP1 | 107 | -0.09054 | 0.3537 |
| ATAD2 | 107 | -0.08628 | 0.3769 |
| PBK | 107 | -0.07484 | 0.4436 |
| CHAF1A | 107 | -0.06855 | 0.483 |
| MCM7 | 107 | -0.06264 | 0.5215 |
| KIF2C | 107 | -0.05897 | 0.5463 |
| CKS1B | 107 | -0.0567 | 0.5618 |
| NOLC1 | 107 | -0.05405 | 0.5803 |
| CBX3 | 107 | -0.05191 | 0.5954 |
| CDK1 | 107 | -0.05151 | 0.5982 |
| PCNA | 107 | 0.009269 | 0.9245 |
| UBE2C | 107 | 0.01763 | 0.8569 |
| TACC3 | 107 | 0.02343 | 0.8107 |
| ADAR | 107 | 0.02974 | 0.761 |
| OAS3 | 107 | 0.07782 | 0.4256 |
| TYMP | 107 | 0.1245 | 0.2013 |
| ISG15 | 107 | 0.1336 | 0.1702 |
| KYNU | 107 | 0.146 | 0.1336 |
| NASP | 107 | 0.1707 | 0.0788 |
| OAS2 | 107 | 0.2444 | 0.0112 |
| STAT1 | 107 | 0.3704 | < 0.0001 |
| NMI | 107 | 0.3741 | < 0.0001 |
| IFI30 | 107 | 0.6258 | < 0.0001 |

**Remarks:** The case number 107 included 11 non-tumor esophageal tissues and 96 ESCC samples.

**Table S4. The correlation between PES1 expression levels and clinicopathologic features of the ESCC patients.**


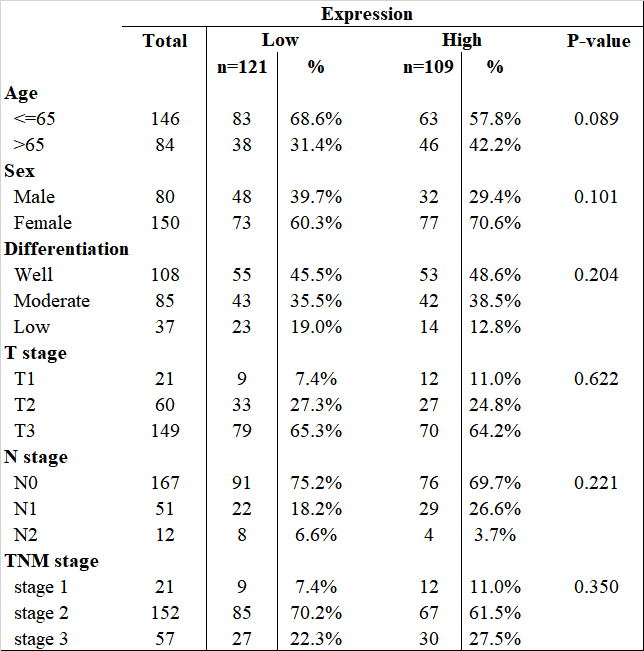


**Table S5. Primary antibodies used in this study.**

| **Antibody** | **Cat No** | **Manufacturer** | **Species** |
| --- | --- | --- | --- |
| PES1 | 13553 | Proteintech | Rabbit |
| PES1 | sc-166300 | Santa Cruz | Mouse |
| ILF3 | 19887 | Proteintech | Rabbit |
| GAPDH | 60004 | Proteintech | Rabbit |
| FLAG | 20543 | Proteintech | Mouse |
| Ki67 | GB111499 | Servicebio | Mouse |
| Granzyme B | 46890 | CST | Rabbit |
| IL15-IHC | ab55276 | Abcam | Rabbit |
| IL15-WB | ab273625 | Abcam | Rabbit |
| CD8 （IHC） | ab209775 | Abcam | Rabbit |
| CD3 （IHC） | ab16669 | Abcam | Rabbit |
| CD4 （IHC） | ab183685 | Abcam | Rabbit |
| NCR1（IHC） | ab233558 | Abcam | Rabbit |
| F4/80（IHC） | ab111101 | Abcam | Rabbit |
| CD45 (Flow) | 103108 | Biolegend | Rat |
| CD3 (Flow) | 100330 | Biolegend | Hamster |
| CD4 (Flow) | 563151 | BD | Rat |
| CD8 (Flow) | 551162 | BD | Rat |
| NK1.1 (Flow) | 108714 | Biolegend | Mouse |
| GZMB (Flow) | 372208 | Biolegend | Mouse |
| CD11B (Flow) | 557396 | BD | Rat |
| F4/80 (Flow) | 565410 | BD | Mouse |
